# Baicalein-Cyclodextrin Inclusion Complexes Nasal Thermosensitive Hydrogel: Bioavailability Improvement and Pharmacokinetic Evaluation in Rats

**DOI:** 10.3390/ph19050781

**Published:** 2026-05-16

**Authors:** Xinyu Ji, Xiali Wei, Zixuan Guo, Ziyang Li, Yuxian Li, Rui Yang, Qingri Jin

**Affiliations:** 1 School of Pharmacy, Hangzhou Medical College, Hangzhou 311399, China18394699835@163.com (X.W.); guozixuan0023@163.com (Z.G.); 13503175219@163.com (Z.L.); 2 Traditional Chinese Medicine Department, Jilin Agricultural Science and Technology College, Jilin 132102, China; liyuxian@jlnku.edu.cn; 3 NMPA Key Laboratory for Quality Research and Evaluation of Pharmaceutical Excipients, National Institutes for Food and Drug Control, Beijing 100050, China

**Keywords:** baicalein, cyclodextrin, thermosensitive hydrogel, nasal administration, bioavailability

## Abstract

**Background**: Baicalein (BA) is a poorly soluble flavonoid with limited oral bioavailability. This study aimed to enhance the solubility and nasal absorption of the compound using a dual-carrier system that combines cyclodextrin inclusion complexes and thermosensitive hydrogels. **Methods**: The inclusion complexes of BA with hydroxypropyl-β-cyclodextrin (HP-β-CD) or sulfobutyl-β-cyclodextrin (SBE-β-CD), namely BA-HP-β-CD and BA-SBE-β-CD, were prepared via solution stirring and characterized by solubility, dissolution, scanning electron microscopy (SEM), Fourier transform infrared spectroscopy (FTIR), X-ray diffraction (XRD), thermogravimetric analysis-differential scanning calorimetry (TG-DSC), and Madin-Darby canine kidney (MDCK) cell permeation. The optimal complexes were incorporated into chitosan/β-glycerophosphate thermosensitive hydrogels (BA/HP-Gel and BA/SBE-Gel), followed by evaluations of gelation properties, in vitro release, and in vivo pharmacokinetics in rats. **Results**: The water solubility of BA-HP-β-CD and BA-SBE-β-CD increased 572 and 582 times, with MDCK permeability enhanced by 5.3 and 2.9 times, respectively. Both hydrogels showed rapid solution-gel transition at nasal temperature and sustained release. Following intranasal administration, BA/HP-Gel and BA/SBE-Gel achieved relative bioavailabilities of 623.5% and 697.8%, respectively, compared with BA-Gel. **Conclusions**: The dual-carrier platform effectively improved BA solubility, permeability, and nasal bioavailability, offering a promising strategy for nasal delivery of poorly soluble drugs.

## 1. Introduction

Baicalein (5,6,7-trihydroxyflavone, BA) is the main active component of *Scutellaria baicalensis Georgi*. It has gained significant attention due to its antioxidant, antibacterial, anti-inflammatory, and antitumor pharmacological activities [1,2,3]. However, according to the Biopharmaceutics Classification System (BCS), BA is classified as a class II drug, characterized by poor water solubility and low bioavailability. These properties significantly limit its widespread clinical application [4]. To address this issue, improving the solubility and bioavailability of BA has become a key research focus.

Cyclodextrins (CDs) are cyclic oligosaccharides. They are composed of glucose units linked by α-1,4-glycosidic bonds. CDs are among the most widely used pharmaceutical excipients. Among them, α-CD, β-CD, and γ-CD are the three most common CDs, containing 6, 7, and 8 glucose units, respectively [5]. β-CD has been widely used due to its relatively low solubility and suitable cavity size (approximately 0.78 nm), which is suitable for many guest molecules [6,7]. Hydroxypropyl-β-cyclodextrin (HP-β-CD) is prepared via the etherification reaction of β-CD with propylene oxide under alkaline conditions [8]. The introduction of hydroxypropyl groups greatly enhances its water solubility (≤600 mg/mL) [9]. Owing to its extremely low toxicity and excellent biocompatibility, HP-β-CD is commonly used for the solubilization and delivery of poorly soluble drugs. Sulfobutylether-β-cyclodextrin (SBE-β-CD) is obtained through the substitution reaction. Under alkaline conditions, hydroxyl groups of β-CD react with 1,4-butane sulfonate, with an average degree of substitution typically ranging from 6.2 to 6.9. Due to the extension of the butyl side chain in the hydrophobic cavity and the high acid dissociation constant, SBE-β-CD exhibits high water solubility (>500 mg/mL). It also has low toxicity, good molecular complexation ability, and excellent hemocompatibility [10]. Unlike HP-β-CD, SBE-β-CD maintains the same ionization state within the pH range of 2.5–8.0, with its surface consistently carrying a negative charge [11].

There are many methods for preparing cyclodextrin inclusion complexes, which can be classified based on different operating principles and technical conditions. They include the aqueous solution method, grinding method, freeze-drying method, solution-stirring method, supercritical carbon dioxide method, spray drying, and microwave irradiation method [12,13]. Among these methods, the solution-stirring method is common and practical. It involves dissolving CDs and the drug in two miscible mixed solvents or in separate solvents to obtain clear solutions. The solvent is then removed by rotary evaporation and vacuum drying to yield a powdery inclusion complex. This method is simple and has a low preparation cost [14]. Cyclodextrin inclusion technology is an efficient and widely used drug delivery system. It forms inclusion complexes through host-guest interactions. This reduces drug crystallinity [15] and improves the physicochemical properties of poorly soluble drugs [16]. CDs have a unique cavity structure, characterized by a hydrophilic outer wall and a hydrophobic cavity [17]. When poorly soluble drug molecules are included in their hydrophobic cavities, the solubility of the drugs in water can be significantly increased [18]. This is particularly important for BCS II and IV drugs, as enhancing solubility is essential for improving drug absorption. However, the actual improvement of bioavailability requires collaborative optimization of the entire absorption pathway [19].

As a non-invasive route of administration, nasal delivery offers several advantages, including rapid absorption, quick onset of action, and avoidance of the first-pass effect [20]. However, the rapid clearance by nasal cilia can lead to a short residence time for conventional solutions in the nasal cavity, thereby limiting the absorption of drugs [21]. To address this issue, thermosensitive hydrogels have emerged as an ideal dosage form due to their unique characteristics. Hydrogels are typically three-dimensional network polymers composed of polymers, proteins, small molecules, or colloids. They provide a multifunctional platform for drug delivery [22]. Thermosensitive hydrogels are a type of intelligent material that remain in a liquid state at low temperatures and rapidly transition to a gel state near body temperature. This “solution-gel” phase change characteristic provides three advantages for nasal drug delivery: accurate delivery, mild administration, and sustained and controlled release. As a novel drug delivery system, thermosensitive hydrogels are characterized by their non-toxicity, high water content, biodegradability, and biocompatibility [23,24]. These hydrogels have been widely applied in various medical fields, including drug delivery [25,26], medical dressings [27,28], and bone tissue engineering [29].

Chitosan (CS) is a natural polysaccharide derived from the deacetylation of chitin [30]. It has excellent antibacterial properties, good biocompatibility, and low toxicity. Moreover, CS can be degraded into non-toxic oligosaccharide products by enzymes in the body [31]. Therefore, hydrogels based on CS are often used as drug-sustained-release carriers [32] and are widely used in biomedical fields. Pentahydrate-β-glycerophosphate sodium (β-GP) can be combined with CS to form temperature-sensitive hydrogels with in situ physical cross-linking properties [33]. In order to further enhance the bioavailability of BA and maximize its pharmacological effects, the cyclodextrin inclusion complexes of BA were formulated into a thermosensitive hydrogel for nasal administration. This hydrogel remained liquid at room temperature and rapidly gelled upon entering the nasal cavity. This prolonged the residence time of the drug in the nasal cavity and achieved a sustained-release effect.

To summarize, the objective of this study is to develop a thermosensitive hydrogel formulation of BA-cyclodextrin inclusion complexes. This preparation uses BA-cyclodextrin inclusion complexes, CS, and β-GP to construct a nasal thermosensitive hydrogel system. The goal is to enhance the water solubility, stability, and bioavailability of BA. The hydrogel system prolongs the residence time of the drug in the nasal cavity, increases the contact time between the drug and nasal mucosa, promotes nasal mucosal absorption, and improves overall bioavailability. This approach offers a new and efficient method for the nasal delivery of poorly soluble drugs. It may provide strong support for the clinical application of related medications.

## 2. Results

### 2.1. Optimization of Baicalein-Cyclodextrin Inclusion Complex Preparation

#### 2.1.1. Orthogonal Experimental Results for Baicalein-Hydroxypropyl-β-Cyclodextrin Inclusion Complex (BA-HP-β-CD)

Based on the experimental results shown in Table 1, the effects of various factors on the preparation of BA-HP-β-CD were as follows: molar ratio of host to guest molecules (A), stirring time (B), amount of water added (C), and inclusion temperature (D). The optimal prescription preparation process, determined based on the range analysis of orthogonal experimental results, was A_3_B_2_C_1_D_3_, corresponding to a molar ratio of 1:3, stirring time of 90 min, water addition of 10 mL, and inclusion temperature of 60 °C.

#### 2.1.2. Orthogonal Experimental Results for Baicalein-Sulfobutylether-β-Cyclodextrin Inclusion Complex (BA-SBE-β-CD)

Based on the experimental results shown in Table 2, the effects of various factors on the preparation of BA-SBE-β-CD were as follows: molar ratio of host to guest molecules (A), amount of water added (C), inclusion temperature (D), and stirring time (B). The optimal prescription preparation process, determined based on the range analysis of orthogonal experimental results, was A_3_B_3_C_3_D_2_, corresponding to a molar ratio of 1:4, stirring time of 120 min, water addition of 40 mL, and inclusion temperature of 30 °C.

### 2.2. Determination of the Optimal Content of Baicalein-Cyclodextrin Inclusion Complexes

The content of BA in the BA-HP-β-CD was 4.95% (*w*/*w*), with an average EE of 73.4%. The content of BA in the BA-SBE-β-CD was 4.03% (*w*/*w*), with an average EE of 90.8%.

### 2.3. Characterization of Baicalein-Cyclodextrin Inclusion Complexes

#### 2.3.1. Solubility Results

As shown in Table 3, the solubility of free BA, BA-HP-β-CD, and BA-SBE-β-CD in pure water was 3.08, 1761.36, and 1811.32 μg/mL, respectively. The results indicated that both cyclodextrin inclusion complexes significantly increased the solubility of BA, with enhancements of 572 and 582 times, respectively. The solubility of the BA-SBE-β-CD was slightly higher than that of the BA-HP-β-CD.

#### 2.3.2. Dissolution Results

**Figure 1:**
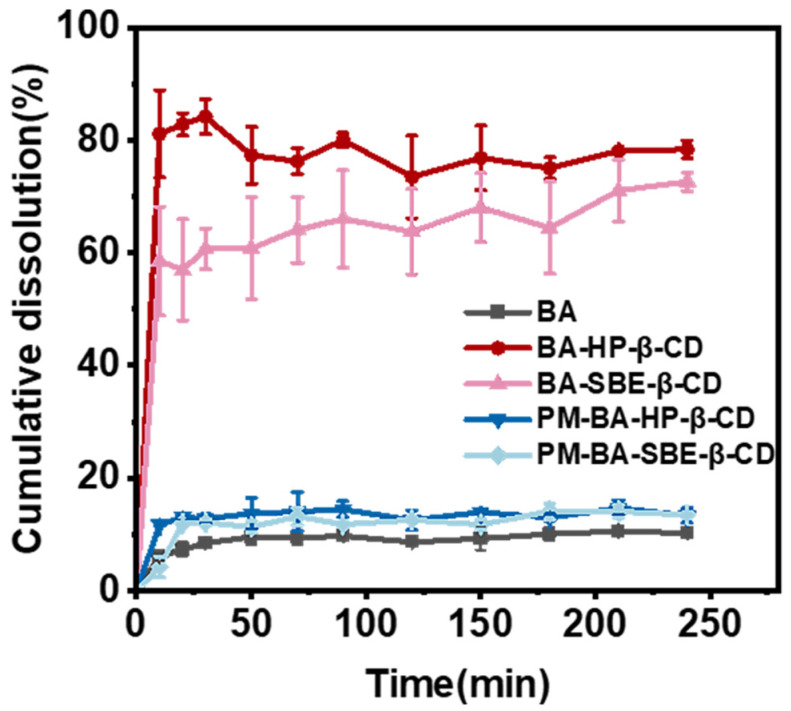
Cumulative dissolution (*n* = 3).

As shown in Figure 1, at 30 min, the cumulative dissolution rates of BA-HP-β-CD and BA-SBE-β-CD reached approximately 80% and 60%, respectively, which were significantly higher than free BA (8.42%) and its physical mixtures (PM), specifically PM-BA-HP-β-CD (12.4%) and PM-BA-SBE-β-CD (12.1%). During the entire 240 min dissolution test, the cumulative dissolution rates of the two inclusion complexes were 7.67 times and 7.10 times that of BA, respectively. These findings suggested that cyclodextrin inclusion technology can significantly improve the dissolution rate of BA. This result can be attributed to the improved solubility of BA due to the inclusion complex formation, which facilitated its release in water. In contrast, PM failed to form stable inclusion structures and therefore had a dissolution behavior similar to that of the free BA. In addition, BA-HP-β-CD showed a higher release rate at the early stage of dissolution, which may have been related to its higher water solubility and dissolution capacity [34].

#### 2.3.3. Scanning Electron Microscope (SEM)

**Figure 2:**
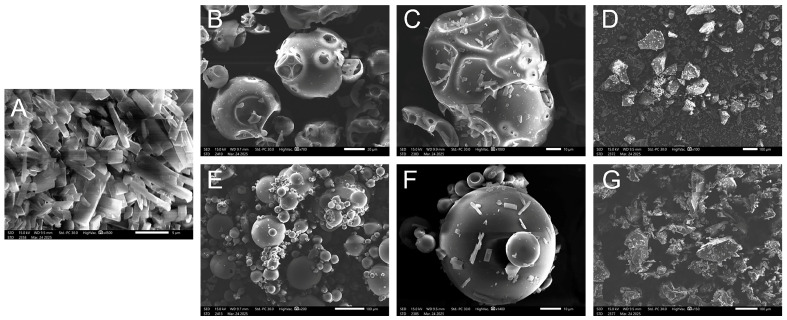
Scanning electron microscope (SEM) of the samples. (**A**) Baicalein (BA), (**B**) Hydroxypropyl-β-cyclodextrin (HP-β-CD), (**C**) Physical mixtures of BA and HP-β-CD (PM-BA-HP-β-CD), (**D**) BA-HP-β-CD, (**E**) Sulfobutyl-β-cyclodextrin (SBE-β-CD), (**F**) Physical mixtures of BA and SBE-β-CD (PM-BA-SBE-β-CD), and (**G**) BA-SBE-β-CD.

SEM was performed on each sample to understand its physical structure and interactions by observing the surface morphology and particle morphology. As shown in Figure 2A, the SEM image of BA revealed a typical crystalline form. It displayed a regular columnar crystal structure [35]. These crystal structures indicated that BA had a highly ordered crystalline arrangement. In contrast, the SEM image of HP-β-CD showed a porous spherical structure (Figure 2B) [36]. The SEM image of SBE-β-CD (Figure 2E) was similar to that of HP-β-CD, but it appeared as a spherical shape. This was due to the introduction of sulfated groups [37]. The SEM images of PM-BA-HP-β-CD (Figure 2C) and PM-BA-SBE-β-CD (Figure 2F) revealed two distinct morphologies. The columnar crystals of BA could be observed on the surfaces of HP-β-CD and SBE-β-CD. This finding indicated a mechanical mixture of the drug and excipients without any change in their respective forms. The SEM images of the BA-HP-β-CD (Figure 2D) and BA-SBE-β-CD (Figure 2G), however, clearly showed an irregular flake-like structure. This finding suggested that the complexes were amorphous, which, to some extent, enhanced the solubility and dissolution rate of BA.

#### 2.3.4. Fourier Transform Infrared Spectroscopy (FTIR)

**Figure 3:**
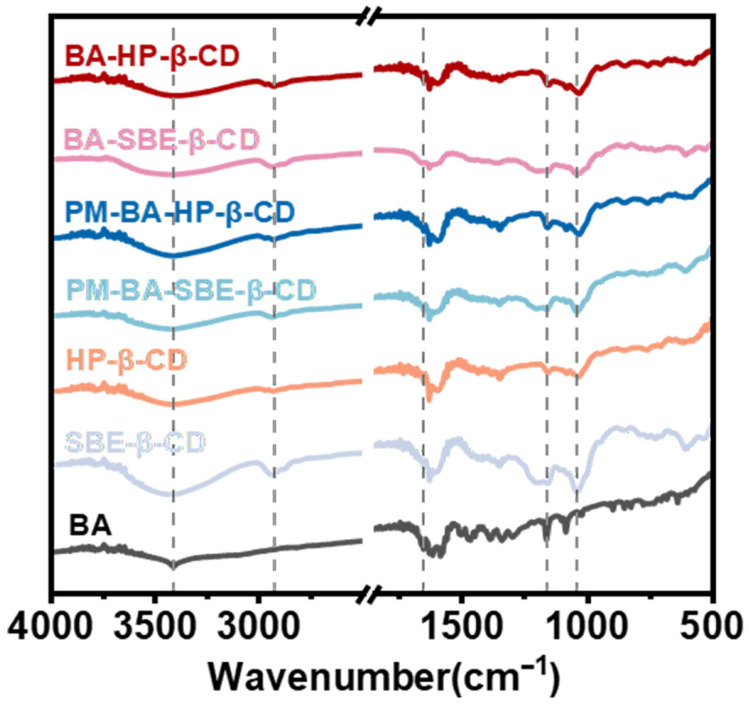
Fourier Transform Infrared Spectroscopy (FTIR) spectra of BA, HP-β-CD, SBE-β-CD, PM-BA-HP-β-CD, PM-BA-SBE-β-CD, BA-HP-β-CD, and BA-SBE-β-CD.

As shown in Figure 3, the spectrum of BA displayed a broad absorption peak at 3411 cm^−1^ corresponding to the hydroxyl group (-OH) and a carbonyl (C=O) stretching vibration peak at 1655 cm^−1^ [38]. Additionally, the stretching vibrations of the aromatic ring were observed at 1617 cm^−1^, 1587 cm^−1^, and 1505 cm^−1^ (C=C) [39]. HP-β-CD exhibited characteristic absorption peaks at 3405 cm^−1^ (-OH), 2929 cm^−1^ (C-H), and 1033 cm^−1^ (C-O) [36]. SBE-β-CD showed a broad and intense absorption peak in the range of 3200–3600 cm^−1^, which was attributed to the stretching vibrations of the -OH groups. These groups mainly originate from the numerous unsubstituted hydroxyl groups in SBE-β-CD [40]. Besides retaining the β-CD framework peaks at 2929 cm^−1^ (C-H) and 1150–1030 cm^−1^, SBE-β-CD introduced new peaks at 1161 cm^−1^ (asymmetric stretching of C-O-C) and 1045 cm^−1^ (stretching of ether linkages) [41].

The spectra of PM-BA-HP-β-CD and PM-BA-SBE-β-CD were simply the superposition of the characteristic peaks of BA and HP-β-CD or SBE-β-CD, respectively. In contrast, the spectra of the BA-HP-β-CD and BA-SBE-β-CD differed from those of the PM.

In the inclusion complexes, the characteristic peaks of BA became broader and less sharp, while the characteristic peaks of HP-β-CD or SBE-β-CD were retained. These changes indicated that the guest molecule BA had entered the cavity of the host molecule, a β-CD derivative, rather than merely undergoing physical adsorption. This result suggested that BA and the β-CD derivatives had formed inclusion complexes.

#### 2.3.5. X-Ray Diffraction (XRD)

**Figure 4:**
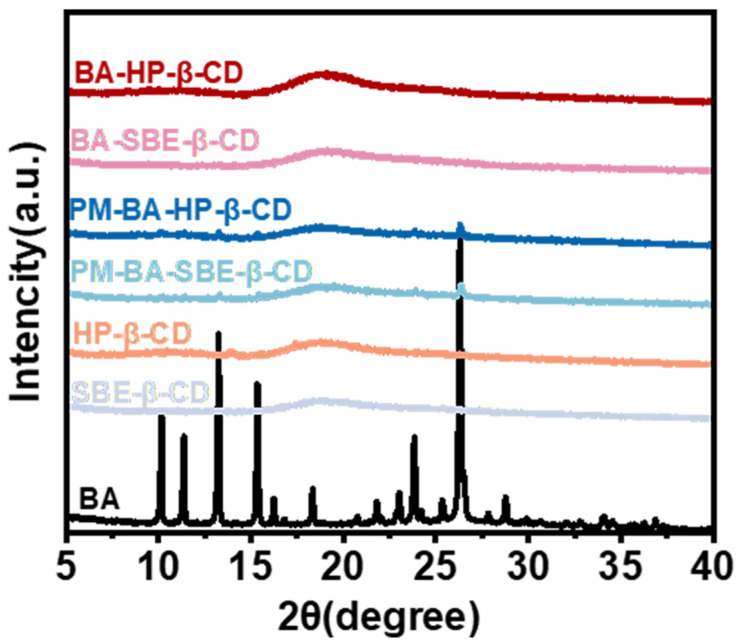
X-Ray Diffraction (XRD) patterns of BA, HP-β-CD, SBE-β-CD, PM-BA-HP-β-CD, PM-BA-SBE-β-CD, BA-HP-β-CD, and BA-SBE-β-CD.

XRD is an important method of studying the crystalline structure of materials. As shown in Figure 4, BA displayed numerous strong and sharp diffraction peaks, indicating a highly ordered crystalline structure. HP-β-CD and SBE-β-CD displayed broader, less-defined peaks that were qualitatively similar to each other, suggesting a comparatively disordered crystalline arrangement [42], but they still possessed some degree of crystallinity.

For the PM samples (PM-BA-HP-β-CD and PM-BA-SBE-β-CD), the diffraction patterns corresponded to a simple superposition of the individual peaks of BA and the respective CDs. These samples were simple physical mixtures. Therefore, BA and the CDs retained their independent crystalline structures. This indicated that physical mixing did not change the crystalline structures of BA and the CDs. However, when BA was successfully included in the cavity of CDs (BA-HP-β-CD and BA-SBE-β-CD), the crystalline structure of BA was disrupted. Its original sharp diffraction peaks completely disappeared, replaced by the broad peaks of CD. This indicated that BA had been successfully included in CD, forming inclusion complexes [43].

#### 2.3.6. Thermogravimetric Analysis-Differential Scanning Calorimetry (TG-DSC)

**Figure 5:**
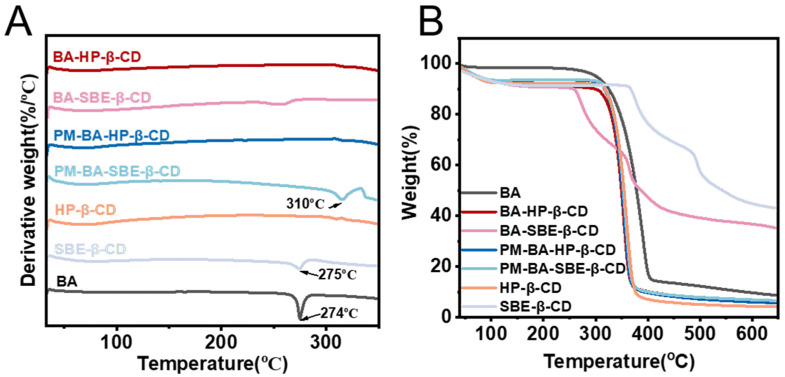
Differential scanning calorimetry (DSC) (**A**) and thermogravimetric analysis (TG) profiles (**B**) of BA, HP-β-CD, SBE-β-CD, PM-BA-HP-β-CD, PM-BA-SBE-β-CD, BA-HP-β-CD, and BA-SBE-β-CD.

As shown in Figure 5A, BA exhibited a distinct endothermic melting peak around 274 °C [44]. The DSC curve of HP-β-CD did not show a significant endothermic peak. In contrast, SBE-β-CD displayed an irregular peak around 275 °C [45]. This peak might be attributed to the endothermic peak resulting from the melting of excipients. The PM samples (PM-BA-HP-β-CD and PM-BA-SBE-β-CD) showed an inconspicuous small peak around 310 °C, which may have resulted from spontaneous amorphization during the DSC analysis. In contrast, neither the BA-HP-β-CD nor the BA-SBE-β-CD displayed the characteristic endothermic peak of BA. This indicated that BA had successfully formed more physically stable inclusion complexes with HP-β-CD or SBE-β-CD. As a result, the BA crystalline structure was disrupted, and its original melting point characteristics were lost.

The TG results further revealed changes in the thermal stability of BA after inclusion complexation (Figure 5B). The weight loss of BA occurred in two stages. The first stage was below 100 °C. It showed a minimal weight loss of only 2%, which corresponded to the loss of water molecules. The second stage was between 251 °C and 426 °C. It showed a substantial mass loss of 83.7%. This indicated poor thermal stability and confirmed the decomposition of BA organic molecules [46,47].

HP-β-CD exhibited a mass loss of 84.6% in the range of 295 °C to 395 °C, showing higher thermal stability, with a decomposition temperature significantly higher than that of BA. SBE-β-CD displayed a two-step weight loss process. The first step was 22.6% between 250 °C and 333 °C. The second step was 25.7% between 333 °C and 462 °C. This two-step weight loss may be related to the thermal cleavage characteristics of the sulfobutyl substituents [48].

Notably, the PM samples (PM-BA-HP-β-CD and PM-BA-SBE-β-CD) exhibited thermal behavior similar to that of BA, with a single-step weight loss of approximately 83.2% in the range of 290 to 395 °C. This indicated that simple physical mixing did not significantly improve the thermal stability of BA.

By contrast, the inclusion complexes showed markedly improved thermal stability. BA-HP-β-CD exhibited a mass loss of 79.7% in the range of 288 to 389 °C. BA-SBE-β-CD displayed a two-step weight loss process. The first step was 22.8% between 229 °C and 332 °C. The second step was 26.9% between 332 °C and 458 °C. Compared with BA, the inclusion complexes exhibited increased thermal decomposition temperatures and reduced mass loss proportions. These results demonstrated that both HP-β-CD and SBE-β-CD can effectively form inclusion complexes with BA, thereby enhancing its thermal stability [49].

#### 2.3.7. Cytotoxicity Assay in Madin-Darby Canine Kidney (MDCK) Cells

**Figure 6:**
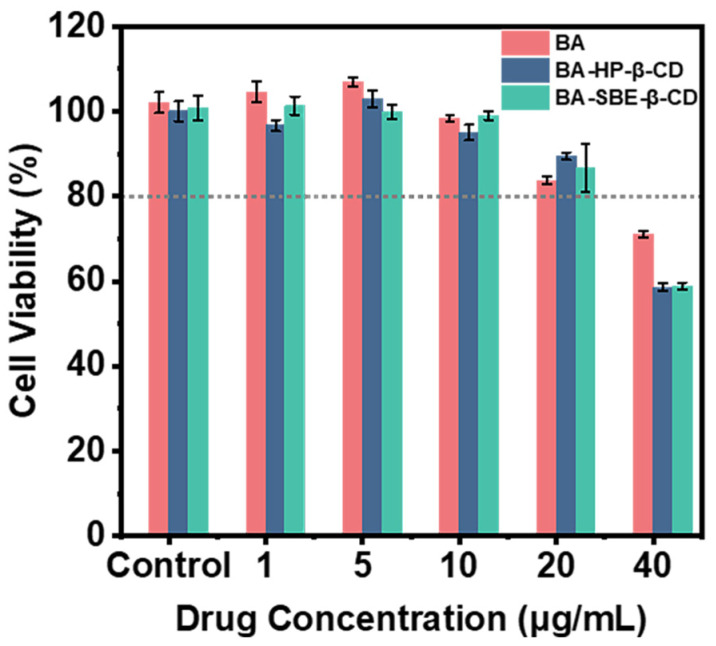
Results of the cell counting kit-8 (CCK-8) assay on Madin Darby canine kidney (MDCK) cells treated with different concentrations of BA, BA-HP-β-CD, and BA-SBE-β-CD (*n* = 3).

The results of the cell counting kit-8 (CCK-8) assay at various drug concentrations are shown in Figure 6. When the concentration of BA was in the range of 0–20 μg/mL, the cell viability of MDCK cells treated with BA, BA-HP-β-CD, and BA-SBE-β-CD was above 80%. This indicated that BA and the two inclusion complexes did not affect cell growth within this concentration range. Based on these results, a drug concentration of 20 μg/mL was selected for the subsequent transmembrane transport experiments.

#### 2.3.8. Transmembrane Transport Study in MDCK Cells

**Figure 7:**
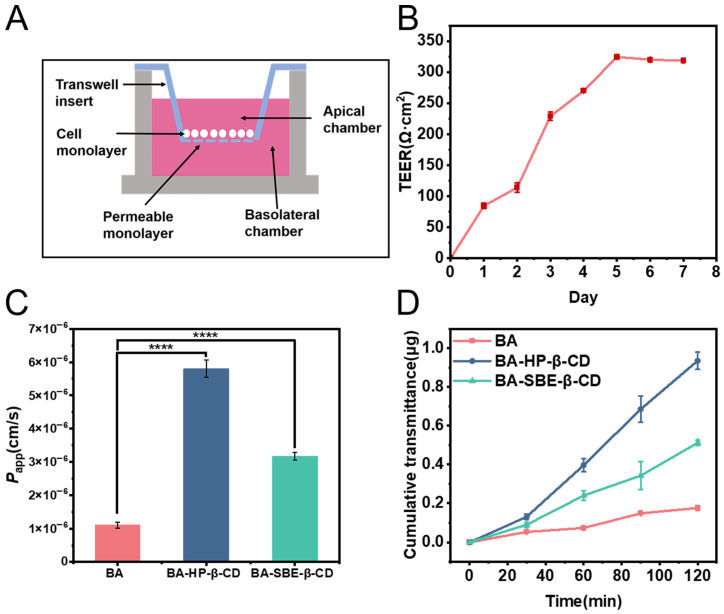
(**A**) Schematic diagram of the Transwell plate structure, (**B**) Transmembrane electrical resistance of MDCK cells in Transwell plates at different days, (**C**) *P*_app_ values, and (**D**) cumulative transport amounts of BA, BA-HP-β-CD, and BA-SBE-β-CD (*n* = 3). **** *p* < 0.0001.

To simulate the absorption and permeation processes of drugs in an in vitro cell model, a single-layer transmembrane transport model was established using MDCK cells [50]. The structural configuration of the Transwell plate used in this model is depicted schematically in Figure 7A. MDCK cells were selected because they exhibit good in vitro and in vivo correlation in nasal absorption studies. Additionally, the time required for MDCK cells to form tight junctions is substantially shorter (7 days) compared to other cell lines (e.g., Caco-2 and Calu-3), enabling rapid formulation screening [51,52]. Furthermore, the main purpose of this study was to screen the membrane permeability of different baicalein formulations.

When the trans-epithelial electrical resistance (TEER) of MDCK cells exceeded 200 Ω·cm^2^, it indicated that tight junctions had been formed. The TEER results of MDCK cells are shown in Figure 7B, with the TEER increasing with culture time and reaching 318 Ω·cm^2^ by day 7. Under the microscope, MDCK cells were observed to form a tight and complete monolayer. This met the criteria for the MDCK cell single-layer transmembrane transport model.

On the 7th day, the TEER of the MDCK cells met the requirements of the experiment, thereby allowing for the progression to the next phase. The cumulative permeation amounts of BA, BA-HP-β-CD, and BA-SBE-β-CD are shown in Figure 7D. The experimental results indicated that, in MDCK cells, the cumulative permeation amounts of the two cyclodextrin inclusion complexes were significantly higher than those of the BA group. The apparent permeability coefficients (*P*_app_) of the two inclusion complexes were also significantly higher than those of the BA group, increasing by 5.3 times and 2.9 times, respectively (Figure 7C). These results suggest that cyclodextrin inclusion technology can enhance the in vitro permeability of BA, which is beneficial for increasing nasal absorption capacity.

### 2.4. Characterization of Baicalein Nasal Thermosensitive Hydrogels

**Figure 8:**
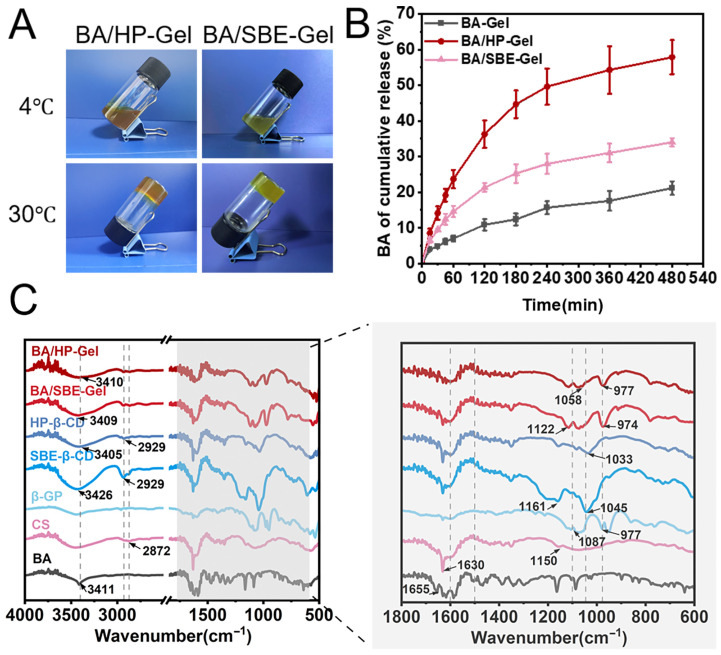
(**A**) Appearance of BA/HP-Gel and BA/SBE-Gel at different temperatures; (**B**) in vitro release curves of BA, BA/HP-Gel, and BA/SBE-Gel (*n* = 3); (**C**) FTIR spectrum of BA, chitosan (CS), pentahydrate-β-glycerophosphate sodium (β-GP), SBE-β-CD, HP-β-CD, BA/HP-Gel, and BA/SBE-Gel.

The physicochemical properties of Baicalein-hydroxypropyl-β-cyclodextrin inclusion complex thermosensitive hydrogel (BA/HP-Gel) and Baicalein-sulfobutylether-β-cyclodextrin inclusion complex thermosensitive hydrogel (BA/SBE-Gel) are summarized in Table 4. The appearances of the two formulations at different temperatures are shown in Figure 8A. The pH values of BA/HP-Gel and BA/SBE-Gel were 7.46 ± 0.02 and 7.38 ± 0.01, respectively, which fell within the acceptable range for nasal administration.

The gelation temperatures of BA/HP-Gel and BA/SBE-Gel were 31.1 ± 0.3 °C and 31.2 ± 0.3 °C, respectively, as determined by the inverted test tube method. These values were slightly higher than those of the blank thermosensitive hydrogel, which may be attributed to the influence of the hydroxyl groups of cyclodextrins on the gelation behavior. Nevertheless, both gelation temperatures were below 34 °C, indicating that the sol–gel transition can be triggered under nasal physiological conditions. The gelation times of BA/HP-Gel and BA/SBE-Gel were 45.0 ± 1.7 s and 58.3 ± 1.5 s, respectively, demonstrating rapid gel formation upon nasal application. The drug content was 5.16 ± 0.02 mg/mL for BA/HP-Gel and 4.67 ± 0.19 mg/mL for BA/SBE-Gel. These results collectively indicated that both BA/HP-Gel and BA/SBE-Gel met the essential requirements for nasal thermosensitive hydrogel formulations [53].

The in vitro release of BA suspension, BA/HP-Gel, and BA/SBE-Gel is shown in Figure 8B. The BA suspension exhibited poor release, with a cumulative release rate of only 21.1% within 480 min. In contrast, the cumulative release rates of BA/HP-Gel and BA/SBE-Gel reached 57.9% and 34.0% within 480 min, respectively. These rates were approximately 2.7 and 1.6 times higher than that of the BA suspension. All three formulations showed a typical sustained-release profile characterized by an initial rapid release phase followed by a slower release phase, without any significant burst release. These results demonstrated that both cyclodextrin-based thermosensitive hydrogels were capable of prolonging the release of BA and maintaining local drug concentrations.

The in vitro release data were fitted to zero-order, first-order, Higuchi, and Ritger-Pappas kinetic models, and the results are summarized in Table 5. The best fits for the in vitro release of BA, BA/HP-Gel, and BA/SBE-Gel were the Higuchi equation, first-order kinetics, and Ritger-Pappas equation, respectively. This indicates that BA/HP-Gel and BA/SBE-Gel had sustained-release effects.

The FTIR spectra of BA/HP-Gel, BA/SBE-Gel, HP-β-CD, SBE-β-CD, β-GP, CS, and BA are shown in Figure 8C. Characteristic absorption peaks for β-GP were observed at 1087 cm^−1^ and 977 cm^−1^. These corresponded to the stretching and bending vibrations of P-O [54]. CS exhibited broad peaks at 1070 cm^−1^ and 1150 cm^−1^, which were assigned to C-O stretching vibrations. A peak was also observed at 1630 cm^−1^, representing the C=O stretching vibration of the amide bond [55]. In addition, CS showed characteristic absorption peaks in the ranges of 3200–3400 cm^−1^, corresponding to -NH_2_ and -OH groups, and a peak at 2872 cm^−1^, corresponding to -CH_2_- [56]. BA showed characteristic peaks at 3411 cm^−1^ (-OH) and 1655 cm^−1^ (C=O). It also showed aromatic ring stretching vibrations at 1617 cm^−1^, 1587 cm^−1^, and 1505 cm^−1^ (C=C). HP-β-CD had characteristic peaks at 3405 cm^−1^ (-OH), 2929 cm^−1^ (C-H), and 1033 cm^−1^ (C-O). SBE-β-CD displayed a broad and intense absorption peak in the range of 3200–3600 cm^−1^ for the stretching vibration of -OH, in addition to peaks at 2929 cm^−1^ (C-H), 1150–1030 cm^−1^ (skeletal peaks), 1161 cm^−1^ (asymmetric stretching of C-O-C), and 1045 cm^−1^ (stretching of ether linkages). The spectra of BA/HP-Gel and BA/SBE-Gel showed characteristic peaks for β-GP, CS, and the respective CDs, while the peaks for BA became broader. This indicated that the hydrogel system was formed by physical interactions and that BA was encapsulated within the hydrophobic cavities of the cyclodextrin molecules.

### 2.5. Pharmacokinetic Study in Rats

**Figure 9:**
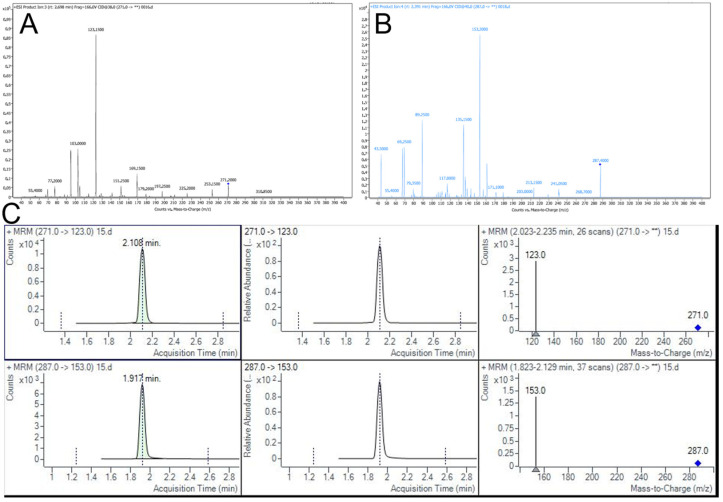
Ion scan spectra of BA (**A**) and luteolin (**B**), and (**C**) multiple reaction monitoring (MRM) quantitative chromatograms and ion information for BA and luteolin. Vertical dashed lines mark the retention time of the target chromatographic peaks, and diamonds denote the mass-to-charge ratio (m/z) of the parent ions. Light green filled peaks represent the quantitative ion chromatograms, and the black curve indicates the chromatographic baseline. Asterisks (**) label the major characteristic fragment ion peaks.

As shown in Figure 9, under the given chromatographic conditions, the retention times of BA and the internal standard were 2.1 min and 1.9 min, respectively. The peak shapes were satisfactory, and there was no interference from endogenous substances, indicating strong specificity of the method. Linear regression analysis was performed on the ratio of the peak areas of the drug to the internal standard, Y (A = A_Baicalein_/A_Luteolin_), versus the concentration of baicalein in plasma (X), with 1/X weighting. The standard curve equation obtained was: Y = 0.06125X + 0.003468 (R^2^ = 0.9964). This indicated that the concentration of baicalein in plasma had a good linear relationship in the range of 10–500 ng/mL.

Before the pharmacokinetic study, the UHPLC-MS/MS method was fully validated. The intra-day and inter-day precisions were evaluated for BA plasma samples at low, medium, and high concentrations (30, 150, and 375 ng/mL). The relative standard deviation (RSD) for both precision measures was below 5% (Table S1). The matrix effect ranged from 104.71% to 107.55%, and the extraction recovery ranged from 101.63% to 104.23%, with RSD within 10% for both parameters (Table S2). In the dilution test, the mean measured concentration was 499.4 ng/mL, which was within 80% of the nominal value, with an RSD of 0.71% (Table S3). Stability studies demonstrated that BA remained stable in plasma under various conditions, including storage at room temperature for 4 h, at 4 °C for 12 h, after three freeze–thaw cycles, and during long-term storage at −80 °C for 7, 14, and 30 d, with no significant impact on quantification (Table S4). Collectively, these results confirm that the established UHPLC-MS/MS method is reliable, accurate, and suitable for the pharmacokinetic study of BA following nasal administration in rats.

**Figure 10:**
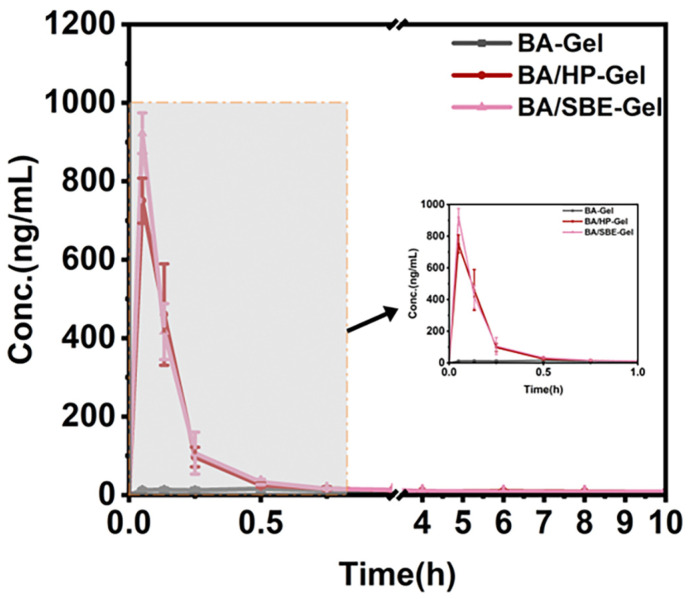
Plasma concentration-time curves after intranasal administration in rats (inset shows the plasma concentration-time curve from 0 to 1 h) (*n* = 4).

The plasma samples from different time points after administration in each group were analyzed using UHPLC-MS/MS. The peak areas were recorded, and the drug concentrations in plasma were calculated using the internal standard method. Non-compartmental analysis was used to calculate the pharmacokinetic parameters. The plasma concentration-time curves and pharmacokinetic parameters of BA-Gel, BA/HP-Gel, and BA/SBE-Gel were compared. The specific results are shown in Figure 10 and Table 6.

As shown in Figure 10 and Table 6, BA/HP-Gel and BA/SBE-Gel exhibited significantly better pharmacokinetic profiles than BA-Gel. In terms of absorption rate, the time to reach maximum plasma concentration (T_max_) for BA-Gel was 0.12 h. In contrast, BA/HP-Gel and BA/SBE-Gel both had a T_max_ of 0.05 h (*p* < 0.0001), indicating that the cyclodextrin inclusion complexes significantly accelerated the nasal absorption rate of BA. Similarly, the maximum plasma concentrations (C_max_) of BA/HP-Gel and BA/SBE-Gel were 750.51 ng/mL and 922.5 ng/mL, respectively. These values were much higher than those of BA-Gel (14.09 ± 4.86 ng/mL) (*p* < 0.0001), demonstrating that cyclodextrin inclusion technology effectively enhanced the nasal absorption efficiency of BA. The elimination half-lives (t_1/2_) of BA/HP-Gel and BA/SBE-Gel were 13.36 h and 14.56 h, respectively, significantly longer than that of BA-Gel (3.46 h) (*p* < 0.01), indicating that the cyclodextrin inclusion complexes prolonged the duration of drug action in the body. Additionally, the mean residence times (MRT_last_) of BA/HP-Gel and BA/SBE-Gel were also significantly higher than that of BA-Gel, further confirming the extended circulation time of the drug in the body.

The AUC_last_ and AUC_0→∞_ of BA/HP-Gel and BA/SBE-Gel were significantly higher than those of BA-Gel (*p* < 0.0001), indicating that the cyclodextrin inclusion complexes not only increased the absorption rate but also significantly enhanced the amount of drug absorbed in the body. The relative bioavailability (F) of BA/HP-Gel and BA/SBE-Gel was 623.5% and 697.8%, respectively, demonstrating that the cyclodextrin inclusion complexes significantly improved the nasal absorption efficiency and bioavailability of BA.

In summary, cyclodextrin inclusion technology significantly improved the nasal absorption efficiency and pharmacokinetic behavior of baicalein, providing a scientific basis for the development of efficient and long-acting nasal formulations of BA.

## 3. Discussion

### 3.1. Physicochemical Characterization of Baicalein-Cyclodextrin Inclusion Complexes

In this study, a dual-carrier delivery system based on cyclodextrin inclusion complexes and thermosensitive hydrogels was successfully constructed. It was designed to address the low bioavailability of BA due to its poor solubility and membrane permeability.

The solubility and dissolution study showed that inclusion complexes formed between BA and HP-β-CD or SBE-β-CD significantly increased their solubility and dissolution rate. This improvement is primarily attributed to the molecular structure of CD, which possesses a hydrophobic cavity and a hydrophilic outer surface. This structure allows BA molecules to partially or completely enter the cavity and form stable inclusion complexes via non-covalent interactions [57]. Notably, the cumulative dissolution rates of the two inclusion complexes reached approximately 80% and 60% at 30 min, respectively, which were markedly higher than that of BA (8.42%) and its PM. This indicated that cyclodextrin inclusion technology is beneficial for improving BA absorption and bioavailability [58,59].

The changes in physicochemical properties further verified the improvement of BA properties. SEM analysis showed that BA exhibited a typical crystalline morphology. In contrast, the inclusion complexes displayed an amorphous state. This morphological change indicated that the crystalline structure of BA was disrupted under the inclusion effect of CD, forming an amorphous state, which generally confers higher solubility and dissolution rates [60]. FTIR spectra showed that the characteristic peaks of BA in the inclusion complexes became broader. At the same time, the inclusion complexes retained the characteristic peaks of CDs. This indicated that BA successfully entered the cavity of CDs rather than undergoing simple physical adsorption. Notably, the PM also showed similar peak broadening to some extent, suggesting that simple mixing may cause partial amorphization. However, XRD results provided clearer evidence: The sharp diffraction peaks of BA completely disappeared in the inclusion complexes. Instead, they were replaced by broad peaks of CDs. This further confirmed the successful formation of inclusion complexes and distinguished them from PM.

The TG-DSC results further revealed the thermal stability of the inclusion complexes. The absence of the characteristic endothermic melting peak of BA in the inclusion complexes indicated the disruption of its crystalline structure and the formation of stable inclusion complexes. However, it should be noted that the PM also did not show a distinct BA peak in DSC. This may be attributed to spontaneous amorphization during the DSC analysis, as indicated by a small inconspicuous peak around 310 °C. This phenomenon does not imply true inclusion complexation. Regarding thermal stability, BA-HP-β-CD exhibited an increased decomposition temperature compared with BA, confirming that HP-β-CD effectively enhanced the thermal stability of BA. In contrast, BA-SBE-β-CD showed a slightly lower onset decomposition temperature (229 °C) than BA (251 °C) in the first step of weight loss. This may be related to the thermal cleavage characteristics of the sulfobutyl substituents. Nevertheless, the second decomposition step of BA-SBE-β-CD occurred at a higher temperature (332–458 °C) than that of BA, suggesting that the inclusion complex still conferred partial protection against thermal degradation. These findings demonstrated that cyclodextrin inclusion enhanced not only the solubility and dissolution rate of BA but also its thermal stability, with HP-β-CD showing superior thermal stabilization compared with SBE-β-CD. Such improvements are beneficial for drug storage and delivery [61].

### 3.2. MDCK Cell Monolayer Transmembrane Transport Study

As shown by the MDCK cell monolayer permeation test, the *P*_app_ of the two inclusion complexes was increased by 5.3 times and 2.9 times compared with BA. These results indicated that cyclodextrin inclusion technology significantly enhanced the cellular permeability of BA, which is crucial for improving nasal absorption. The core mechanism of nasal drug delivery is the penetration of the drug through the nasal mucosa into the systemic circulation. CD encapsulates BA within its hydrophobic cavity to form stable inclusion complexes, which significantly improve the water solubility of BA and enhance its dispersion in nasal mucus. Additionally, CD may temporarily alter the permeability of the nasal mucosal cell membrane or modulate tight junctions between cells, thereby facilitating the passage of BA across the cell membrane into the bloodstream [62,63,64].

### 3.3. Thermosensitive Hydrogel Properties

The thermosensitive hydrogel exhibited distinct sustained-release properties in vitro, with cumulative release rates of BA/HP-Gel and BA/SBE-Gel reaching 57.9% and 34.0% within 480 min, respectively, both significantly higher than that of BA suspension (21.1%). The release profiles followed a typical biphasic pattern with no significant burst release. BA/HP-Gel showed faster release than BA/SBE-Gel. This difference may be due to differences in cyclodextrin-gel matrix interactions or gel viscosity. After 240 min, the similar slopes suggested a transition to dissolution-controlled release. A major limitation of the dialysis method employed in this study is its propensity to create non-sink conditions, as the membrane surface area restricts the transport of released drug from the inner compartment to the release medium [65,66]. Due to the very low aqueous solubility of BA, strict sink conditions were not confirmed in this study. Nevertheless, the release study was performed under the same conditions across all formulations, allowing for a meaningful comparative evaluation of their relative release behaviors. This strategy has been widely accepted in dialysis-based in vitro release studies involving poorly soluble drugs [67,68]. These results suggest that both hydrogels can prolong BA release, and upon intranasal administration, the “solution-gel” transition may reduce mucociliary clearance, extend mucosal contact time, and form a sustained-release reservoir, potentially enhancing nasal absorption [69,70].

Although direct nasal irritation and ciliotoxicity assessments were not performed in this study, the biocompatibility and safety of the CS/β-GP thermosensitive hydrogel system have been documented. CS and its derivatives have been shown to possess excellent biocompatibility, low toxicity, and good mucoadhesive properties, making them suitable for nasal drug delivery applications [71]. Furthermore, β-GP is an organic mineral naturally found in the body and has been approved for clinical use as a phosphate source [72]. Specifically, chitosan-based thermosensitive hydrogels have been reported to exhibit no apparent cytotoxicity after nasal administration and no irritation to the nasal mucosa [69,73]. Therefore, despite the lack of direct experimental evaluation in this study, the established safety profile of the CS/β-GP system provides indirect support for the biocompatibility of the developed formulations.

### 3.4. Pharmacokinetic Study

In the rat pharmacokinetic study, BA/HP-Gel and BA/SBE-Gel exhibited significantly better pharmacokinetic profiles than BA-Gel. Specifically, both formulations achieved a shorter T_max_, a higher C_max_, and prolonged t_1/2_ and MRT_last_. Moreover, both formulations showed significantly increased AUC_last_ and AUC_0→∞_, with F reaching 623.5% and 697.8%, respectively. These results demonstrated that the combination of cyclodextrin inclusion complexes and thermosensitive hydrogels significantly enhanced the bioavailability of BA.

An interesting observation emerged from these data. Although BA-HP-β-CD exhibited a superior cumulative dissolution rate (1.3 times) and in vitro cell permeability (1.8 times) compared with BA-SBE-β-CD, the final BA/HP-Gel formulation showed slightly lower in vivo bioavailability (623.5%) than BA/SBE-Gel (697.8%). Several mechanistic factors may explain this apparent discrepancy. First, as shown in Figure 8B, BA/HP-Gel exhibited faster and more complete release (57.9% at 480 min) than BA/SBE-Gel (34.0% at 480 min). While rapid release is generally favorable for absorption, it may also generate transient supraphysiological drug concentrations at the nasal mucosal surface that exceed the absorptive capacity of the epithelium. This mismatch can result in premature clearance by nasal mucociliary transport before complete absorption occurs, effectively reducing the fraction of the dose absorbed [74]. Second, the negative charge of SBE-β-CD may confer enhanced mucoadhesion and transient permeation-enhancing effects on the nasal epithelium. It may interact with membrane cholesterol and phospholipids, disrupting membrane fluidity and facilitating drug permeation [75]. This would lead to prolonged residence time and greater overall absorption, compensating for its lower solubility and permeability in vitro. Thus, BA/SBE-Gel achieved higher bioavailability despite inferior in vitro performance, highlighting that in vivo absorption cannot be directly predicted from in vitro properties alone.

### 3.5. Limitations

Several limitations of this study should be acknowledged. First, no formal stability assessments (short-term, long-term, and accelerated stability testing) were performed on the developed formulations. Although thermal analysis confirmed drug-excipient compatibility, it does not replace real-time stability data. Second, the mechanistic explanations for the discrepancy between in vitro and in vivo results remain speculative due to the lack of direct experimental evidence. Third, key nasal formulation evaluations such as mucoadhesion, mucociliary clearance, and nasal irritation/ciliotoxicity were not performed. Fourth, the use of MDCK cells does not fully replicate the complex features of human nasal epithelium. Fifth, a comprehensive rheological characterization of the hydrogel was not performed.

Future studies should therefore focus on the following aspects: (1) conducting formal stability studies to evaluate the stability of the formulations; (2) evaluating mucociliary clearance, mucoadhesion, and nasal irritation of the thermosensitive hydrogel system; (3) employing more physiologically relevant nasal epithelial models; and (4) conducting comprehensive rheological characterization of the thermosensitive hydrogel.

## 4. Materials and Methods

### 4.1. Materials

HP-β-CD and SBE-β-CD were purchased from Zhiyuan Biotechnology Co., Ltd., Binzhou, China. Chitosan was obtained from Xinfang Pharmaceutical Co., Ltd., Changsha, China. Phosphate-buffered saline (PBS), penicillin/streptomycin solution, 0.25% Trypsin-EDTA, DMEM medium, and Hank’s balanced salts solution (HBSS) were sourced from Gibco, Grand Island, NY, USA. Fetal bovine serum was purchased from Yisheng Biotechnology, Shanghai, China. The CCK-8 kit was obtained from Biyuntian Biotechnology Co., Ltd., Shanghai, China. Additionally, baicalein, KBr, sodium ascorbate, β-glycerophosphate sodium pentahydrate (≥95%), acetic acid, formic acid (HPLC ≥ 99%), acetonitrile, luteolin, Tween 80, potassium dihydrogen phosphate, dipotassium hydrogen phosphate, and sodium hydroxide (95%) were all purchased from Macklin Biochemical Co., Ltd., Shanghai, China. The 0.4 μm Transwell inserts and 96-well plates were obtained from a selected supplier. The MDCK cell line was sourced from the American Type Culture Collection (ATCC, Manassas, VA, USA) cell bank.

### 4.2. Methods

#### 4.2.1. Preparation of Baicalein Cyclodextrin Inclusion Complexes

The solution-stirring method [76] was employed to prepare the baicalein-cyclodextrin inclusion complexes. BA and CDs (HP-β-CD and SBE-β-CD) were accurately weighed according to a specific molar ratio. A cyclodextrin solution was prepared by dissolving the cyclodextrin in distilled water. BA was dissolved in an appropriate amount of anhydrous ethanol, which was then slowly injected via a syringe into the cyclodextrin solution, stirred at a constant temperature. The mixture was stirred for a specified duration. The anhydrous ethanol was removed by rotary evaporation at 55 °C. The resulting product was dried overnight in an oven at 50 °C, ground in a mortar, and sieved through an 80-mesh screen to obtain the baicalein-cyclodextrin inclusion complexes.

#### 4.2.2. High-Performance Liquid Chromatography (HPLC) Conditions for Baicalein

Using an Agilent 1260 high-performance liquid chromatograph and a Welch Ultimate C18 column (4.6 × 150 mm, 5 μm), the mobile phase is methanol: 0.1% formic acid aqueous solution = 6:4 (the mobile phase is filtered by a 0.45 μm microporous membrane); the detection wavelength is 275 nm; the column temperature is 35 °C; the flow rate is 1.0 mL/min; and the injection volume is 20 μL.

#### 4.2.3. Determination of Encapsulation Efficiency of Baicalein-Cyclodextrin Inclusion Complexes

BA-HP-β-CD and BA-SBE-β-CD (30 mg of each) were accurately weighed and placed into a 10 mL brown volumetric flask. Distilled water was added up to near the calibration mark, followed by ultrasonication for 10 min. After cooling to room temperature, the volume was adjusted with distilled water, mixed thoroughly, and filtered through a 0.45 μm membrane filter. The filtrate was appropriately diluted with the mobile phase and analyzed under the specified chromatographic conditions, with the peak area recorded as W_1_. Separately, 30 mg each of BA-HP-β-CD and BA-SBE-β-CD were weighed and transferred into a 10 mL brown volumetric flask. Methanol was added to dissolve the sample, followed by ultrasonication for 10 min. After cooling to room temperature, the volume was adjusted with methanol, mixed thoroughly, and filtered through a 0.45 μm membrane filter. The filtrate was appropriately diluted with the mobile phase and analyzed under the specified chromatographic conditions, with the peak area recorded as W_2_.

Encapsulation efficiency (EE, %) = W_1_/W_2_ × 100%

#### 4.2.4. Optimization of Baicalein-Cyclodextrin Inclusion Complexes

Orthogonal experiments can identify the key factors affecting the formation of inclusion complexes with the minimum number of trials [36]. Using the molar ratio of host to guest molecules, stirring time, amount of water added, and inclusion temperature as variables and the encapsulation efficiency as the response value, an L_9_ (3^4^) orthogonal experimental design was employed for analysis to screen the optimal conditions for preparing the inclusion complexes. The specific factors and levels affecting the formation of BA-HP-β-CD and BA-SBE-β-CD are shown in Table 7 and Table 8, respectively.

#### 4.2.5. Physicochemical Characterization of Baicalein-Cyclodextrin Inclusion Complexes

##### Preparation of Baicalein Cyclodextrin Physical Mixtures

BA was accurately weighed and mixed with HP-β-CD and SBE-β-CD in a mortar at molar ratios of 1:3 and 1:4, respectively. The components were thoroughly ground to ensure uniform mixing, yielding the PM (PM-BA-HP-β-CD and PM-BA-SBE-β-CD).

##### Solubility Study

Accurately weigh 30 mg of BA, BA-HP-β-CD, and BA-SBE-β-CD (each equivalent to 30 mg of BA) separately and transfer them into centrifuge tubes containing 10 mL of distilled water. The solutions were shaken at 37 °C for 24 h in a constant-temperature shaker, then filtered through a 0.45 μm membrane filter. The filtrates were appropriately diluted with the mobile phase, and the BA content in distilled water was determined by HPLC to calculate its solubility.

##### Dissolution Study

The dissolution profiles of BA, BA-HP-β-CD, BA-SBE-β-CD, PM-BA-HP-β-CD, and PM-BA-SBE-β-CD were evaluated through dissolution studies using an RC-806D dissolution apparatus (TDTF, Tianjin, China). The paddle method was employed with 500 mL of distilled water (containing 0.01% sodium ascorbate) as the dissolution medium, maintained at 37 ± 0.5 °C with a stirring speed of 50 rpm.

10 mg of BA, BA-HP-β-CD, BA-SBE-β-CD, PM-BA-HP-β-CD, and PM-BA-SBE-β-CD (each equivalent to 10 mg of BA) were placed in the dissolution vessels. Samples of 5 mL were withdrawn at 10, 20, 30, 50, 70, 90, 120, 150, 180, 210, and 240 min, respectively. Each sample was filtered through a 0.45 μm membrane filter, and the same volume of dissolution medium at the same temperature was replenished. The filtrates were analyzed by HPLC to determine the content of BA, and the cumulative dissolution rate was calculated.

##### Scanning Electron Microscope (SEM)

BA, HP-β-CD, SBE-β-CD, PM-BA-HP-β-CD, PM-BA-SBE-β-CD, BA-HP-β-CD, BA-HP-β-CD, and BA-SBE-β-CD were, respectively, weighed on the sample holder with conductive tape. After gold-coating, the samples were analyzed using a JSM-IT200 scanning electron microscope (JEOL, Tokyo, Japan) at an accelerating voltage of 15.0 kV, and images were captured using SEM equipped with In Touch Scope™ software (2.0).

##### Fourier Transform Infrared Spectroscopy (FTIR)

BA, HP-β-CD, SBE-β-CD, PM-BA-HP-β-CD, PM-BA-SBE-β-CD, BA-HP-β-CD, and BA-SBE-β-CD were weighed and mixed in an agate mortar. Infrared absorption spectra were scanned using A Nicolet iS5 spectrometer (Thermo Fisher Scientific, Waltham, MA, USA) over a wavenumber range of 400 to 4000 cm^−1^, with a resolution of 4 cm^−1^. The spectra were analyzed using OMNIC Paradigm software (OMNIC 9.3.32), and the results were plotted using Origin 2024 software [36].

##### X-Ray Diffraction Analysis (XRD)

XRD analysis was performed on an XRD-6000 (Shimadzu, Kyoto, Japan) diffractometer equipped with a Cu-Kα radiation source. The diffractometer was operated at 40 kV and 30 mA. Data were collected over a 2θ range of 5–35° at a scanning speed of 2°/min. Sample data were collected, and the XRD spectra were recorded accordingly [77].

##### Thermogravimetric Analysis-Differential Scanning Calorimetry (TG-DSC)

The samples were analyzed using a Mettler TG-DSC analyzer (Mettler Toledo TGA/DSC2 LF1600, Greifensee, Switzerland) under simultaneous thermogravimetric conditions. Approximately 10 mg of each sample, including BA, HP-β-CD, SBE-β-CD, PM-BA-HP-β-CD, PM-BA-SBE-β-CD, BA-HP-β-CD, and BA-SBE-β-CD, were weighed and analyzed. The operating conditions were as follows: heating rate of 10 °C/min, nitrogen purge flow rate of 50 mL/min, protective nitrogen flow rate of 60 mL/min, and temperature scanning range of 30–700 °C [78].

#### 4.2.6. Transmembrane Transport Study of Baicalein and Its Cyclodextrin Inclusion Complexes Based on the MDCK Cell Model

##### MDCK Cytotoxicity Test

The cytotoxicity of BA and its cyclodextrin inclusion complexes towards MDCK cells was assessed using the CCK-8 assay. The procedure was as follows: Accurate amounts of BA, BA-HP-β-CD, and BA-SBE-β-CD were weighed and dissolved in DMSO to prepare stock solutions at concentrations of 1, 5, 10, 20, and 40 mg/mL. These stock solutions were then diluted with complete culture medium to obtain drug solutions at concentrations of 1, 5, 10, 20, and 40 μg/mL.

MDCK cells were seeded into a 96-well plate at a density of 5 × 10^3^ cells per well (100 μL/well) and incubated at 37 °C with 5% CO_2_ and 95% relative humidity for 24 h. Upon reaching 70–80% confluence, the culture medium was removed and replaced with fresh medium containing various concentrations of BA, BA-HP-β-CD, and BA-SBE-β-CD (100 μL/well). Negative control (cells without drugs) and blank control (wells without cells) were also prepared. After 24 h of incubation, the 96-well plate was removed, the medium was aspirated and discarded, and each well was washed twice with PBS. Subsequently, 10% (*v*/*v*) CCK-8 reagent was added to each well, followed by incubation at 37 °C for 0.5 h. The absorbance (OD) at 450 nm was then quickly measured using a microplate reader. The cell viability [79] was calculated as follows:

Cell viability (%) = (OD_sample_ − OD_blank_)/(OD_control_ − OD_blank_)

Here, OD_sample_ represents the absorbance of the wells treated with the drug, OD_control_ represents the absorbance of the negative control wells (cells without drugs), and OD_blank_ represents the absorbance of the blank control wells (wells without cells).

When the cell survival rate is ≥80%, it can be considered that the drug with this concentration has no toxic effect on cells.

##### MDCK Cell Transmembrane Transport Study

MDCK cells were seeded at a density of 5 × 10^4^ cells per well in a 12-well Transwell plate. The apical side (AP) was filled with 0.5 mL of cell suspension, while the basolateral side (BL) was filled with 1.5 mL of complete culture medium. The cells were cultured in a cell incubator at 37 °C with 5% CO_2_ and 95% relative humidity for 7 days. After seeding, the medium was changed daily by replacing the complete culture medium (0.5 mL in AP and 1.5 mL in BL). During medium change, the lower layer was aspirated first, followed by the upper layer, and then the upper layer was replenished with 0.5 mL of fresh medium before adding 1.5 mL to the lower layer. The integrity of the cell monolayer was evaluated using an epithelial voltmeter to measure the trans-epithelial electrical resistance (TEER; RE1600, Beijing Jingong Hongtai Technology Co., Ltd., Beijing, China). When the TEER value reached 200 Ω·cm^2^, the cell monolayer was considered to be dense and intact and was ready for subsequent transport studies [80].

The established MDCK cell monolayer transport model was washed with HBSS buffer and then equilibrated in the incubator for 30 min. Subsequently, the HBSS solution from both the AP and BL sides of the Transwell plate was aspirated. The AP side was then loaded with 0.5 mL of the drug solution (20 μg/mL), while the BL side was filled with 1.5 mL of HBSS buffer. The plate was incubated in the incubator, and at 30, 60, 90, and 120 min, 200 μL samples were withdrawn from the BL side and immediately replaced with an equal volume of blank HBSS. The samples were analyzed by HPLC. The Apparent Permeability Coefficient (*P*_app_) [52] was calculated using the following formula:

*P*_app_ = (dQ/dt)/(A·C_0_)

In the formula, dQ/dt represents the permeation rate per unit time; A is the surface area of the polycarbonate membrane (1.12 cm^2^); and C_0_ is the initial concentration of the drug (μg/mL).

#### 4.2.7. Characterization of Baicalein Nasal Thermosensitive Hydrogel

##### Preparation and Optimization of Nasal Thermosensitive Hydrogel

CS powder was accurately weighed and dissolved in a 0.1 mol/L acetic acid solution (prepared by diluting 572 μL of glacial acetic acid to 100 mL and filtering through a 0.22 μm membrane filter) at 4 °C. β-GP was dissolved in distilled water, filtered through a 0.45 μm membrane filter, and stored at 4 °C. Under ice bath conditions, the β-GP solution was slowly added dropwise to the CS solution with magnetic stirring for 30 min to obtain a clear precursor solution, which was then stored at 4 °C [81].

The formulation was systematically optimized using single-factor experiments followed by a Box–Behnken design response surface methodology. The optimization parameters included CS concentration (1.4–3.0%, *w*/*v*), β-GP concentration (40–60%, *w*/*v*), and β-GP/CS volume ratio (1:9–1:1), with gelation temperature (target 30.0 ± 0.5 °C) as the response variable. Based on the optimization results, the optimal blank hydrogel formulation was determined. Detailed optimization data are provided in the Supplementary Information (Tables S5–S7). The optimal blank hydrogel was then loaded with BA-HP-β-CD and BA-SBE-β-CD to obtain BA/HP-Gel and BA/SBE-Gel, respectively.

##### Preparation of Baicalein Nasal Thermosensitive Hydrogel

Based on the results of the preliminary single-factor experiments, the optimal formulation for the baicalein nasal thermosensitive hydrogel was determined to be a CS concentration of 1.8% (*w*/*v*), a β-GP concentration of 55% (*w*/*v*), and a β-GP to CS volume ratio of 1:1. The specific preparation process is as follows: First, the baicalein-cyclodextrin inclusion complex was dissolved in an appropriate amount of 0.1% acetic acid aqueous solution and stirred until uniform. Then, CS powder was added to achieve the desired concentration, and the mixture was stirred again until uniform. The solution was stored at 4 °C for later use. An accurate amount of β-GP was weighed into a vial, dissolved in distilled water, filtered through a 0.45 μm membrane filter, and stored at 4 °C. In an ice bath, the β-GP solution was slowly added dropwise to the CS solution containing the corresponding baicalein-cyclodextrin inclusion complex (BA-HP-β-CD or BA-SBE-β-CD), and the mixture was stirred with a magnetic stirrer for 30 min. The resulting hydrogels (BA/HP-Gel and BA/SBE-Gel) were stored at 4 °C for further use.

##### Drug Content Determination

1 mL of each of three batches of BA/HP-Gel or BA/SBE-Gel was transferred into separate 10 mL volumetric flasks. Methanol was added, and the mixtures were ultrasonicated for 10 min. The resulting solutions were filtered through a 0.45 μm membrane filter, diluted with the mobile phase, and then analyzed by HPLC under the chromatographic conditions.

##### Appearance, pH, Gelation Temperature, and Gelation Time of Baicalein Nasal Thermosensitive Hydrogel

For each of the three batches of BA/HP-Gel and BA/SBE-Gel, an appropriate amount was taken and placed in a colorless, transparent vial. The appearance of the hydrogels at 4 was visually inspected. An appropriate amount of BA/HP-Gel and BA/SBE-Gel was also taken and placed in a beaker. The pH of the hydrogels was measured using a pH meter calibrated with pH buffer solutions.

The gelation temperatures of BA/HP-Gel and BA/SBE-Gel were determined using the inverted test tube method [82]. For each of the three batches, 2 mL of BA/HP-Gel and BA/SBE-Gel were placed into 5 mL centrifuge tubes, which were then immersed in a constant-temperature water bath for heating. The temperature was increased by 1 °C every 2 min. The tubes were periodically removed and quickly tilted at a 90° angle to observe the flow of the sample. The temperature at which the sample ceased to flow was recorded as the gelation temperature. Additionally, the gelation time was measured for three batches of BA/HP-Gel and BA/SBE-Gel. For this purpose, 2 mL of each hydrogel was placed into 5 mL centrifuge tubes and immersed in a water bath maintained at 34 °C. A stopwatch was immediately started. The time taken for the sample to transition from a freely flowing liquid to a non-flowing semisolid was recorded as the gelation time.

##### Fourier Transform Infrared Spectroscopy (FTIR)

BA, CS, β-GP, and the lyophilized BA/HP-Gel and BA/SBE-Gel were mixed with dry KBr powder at a mass ratio of 1:200. The mixtures were thoroughly ground in an agate mortar to ensure homogeneity. An appropriate amount of each sample was placed into a die and compressed into transparent pellets using a manual pellet press. Infrared absorption spectra were recorded in the wavenumber range of 400 to 4000 cm^−1^ with a resolution of 4 cm^−1^. The spectra were analyzed using OMNIC Paradigm software (OMNIC 9.3.32), and the results were plotted using Origin 2024 software.

##### In Vitro Release Study

Preparation of artificial nasal fluid: accurately weigh 13.6 g of potassium dihydrogen phosphate (containing 0.2% sodium ascorbate and 0.2% Tween 80). The substance was dissolved in an appropriate amount of water, the pH was adjusted to 6.4 with 0.1 mol/L NaOH solution (a pH value equivalent to that of physiological nasal mucus [83]), and the resulting solution was then diluted with distilled water to a final volume of 2 L.

The dialysis method was used for the in vitro release of BA [68]. For the in vitro release study, fill a beaker with 500 mL of the above artificial nasal fluid (pH 6.4) and place the beaker in a water bath set at 34 °C (simulating nasal cavity temperature) with a stirring speed of 50 rpm. Separately load BA/HP-Gel, BA/SBE-Gel, and BA suspension (each containing 5 mg of BA) into dialysis bags (molecular weight cut-off: 3500 Da), seal the bags, and submerge them completely into the beaker containing the artificial nasal fluid. At time points of 15, 30, 45, 60, 120, 180, 240, 360, and 480 min, withdraw 1 mL samples from the beaker and immediately replace the withdrawn volume with an equal amount of pre-warmed (34 °C) fresh artificial nasal fluid. Filter each sample through a 0.45 μm filter membrane, and analyze the filtrate by HPLC under the previously described chromatographic conditions to determine the concentration of BA. Calculate the cumulative release rate of BA, and plot the in vitro release curve with time (min) on the *x*-axis and cumulative release rate (%) on the *y*-axis. Each sample was performed in triplicate.

#### 4.2.8. Pharmacokinetic Studies in Rats

##### Plasma Sample Preparation

After complete thawing, plasma samples were vortexed for 5 min. A volume of 60 μL of plasma was transferred to a 1.5 mL centrifuge tube, followed by the addition of 10 μL of luteolin (5 μg/mL) and 20 μL of 0.5 mg/mL sodium ascorbate solution. After mixing thoroughly, 120 μL of acetonitrile (containing 2% formic acid) was added. The mixture was vortexed for 10 min and then centrifuged at 13,000 rpm for 10 min at 4 °C. The supernatant was directly injected for analysis.

##### Detection of Baicalein in Rat Plasma by Ultra-High Performance Liquid Chromatography-Tandem Mass Spectrometry (UHPLC-MS/MS)

Plasma samples were analyzed using UHPLC-MS/MS (Agilent 6495C, Santa Clara, CA, USA). An Agilent SB-C18 column (2.1 mm × 100 mm, 2.7 μm) was used at a column temperature of 40 °C with a flow rate of 0.3 mL/min and an injection volume of 5 μL. Gradient elution was performed with mobile phase A (0.1% formic acid in water) and mobile phase B (acetonitrile): 0–1.3 min, 90% A and 10% B; 1.3–3.5 min, 10% A and 90% B; 3.5–4.0 min, 90% A and 10% B. MS/MS analysis was conducted on an Agilent 6495C triple quadrupole mass spectrometer using electrospray ionization in positive ion mode. The quantification ion transitions were BA at m/z 271.0 → 123.0 (collision energy 38 V) and the internal standard luteolin at m/z 288.0 → 153.0 (collision energy 40 V).

The detailed procedures for the UHPLC-MS/MS method validation are provided in the Supplementary Information.

##### Animals

Twelve healthy male SD rats (350 ± 10 g) were purchased from the Experimental Animal Center of Hangzhou Medical College and housed in a specific pathogen-free animal facility. All animal experiments were conducted in accordance with the Regulations on the Administration of Laboratory Animals and were approved by the Animal Care and Use Committee of the Ministry of Science and Technology. The ethical approval number for the laboratory animal use is “202502012”. Before treatment, the rats were housed under standard laboratory conditions (22 ± 2 °C, 12 h light/12 h dark cycle) with free access to food and water.

##### Pharmacokinetic Study

The 12 SD rats were randomly divided into three groups (*n* = 4 per group): BA-Gel, BA/HP-Gel, and BA/SBE-Gel. The rats were fasted for 12 h before the experiment but allowed free access to water. After anesthesia with pentobarbital sodium, the rats were fixed on a rat board, and femoral artery and vein cannulation surgeries were performed. The formulations were administered intranasally at a dose of 2 mg/kg using a pipette. Blood samples were collected from the femoral artery at 0.05, 0.133, 0.25, 0.5, 0.75, 1, 2, 4, 6, 8, and 10 h post-dosing, with normal saline administered through the femoral vein. The collected blood samples were placed in heparinized centrifuge tubes, centrifuged at 13,000 rpm for 10 min, and the plasma supernatant was transferred to clean centrifuge tubes and stored at −80 °C until analysis.

Non-compartmental analysis was performed on the plasma concentration-time data of each animal using WinNonlin software to calculate the main pharmacokinetic parameters. The relative bioavailability (F) was calculated using the following formula: F (%) = AUC_T_/AUC_R_ × 100%.

AUC_T_ is the area under the plasma concentration-time curve of the test formulation; AUC_R_ is the area under the plasma concentration-time curve of the reference formulation.

#### 4.2.9. Data Processing and Statistical Analysis

Statistical analysis and graphing were performed using GraphPad Prism 9.0 and Origin 2024 software. All data are presented as mean ± standard deviation (Mean ± SD). The statistical comparison of results was carried out using one-way analysis of variance (ANOVA), followed by Dunnett’s post hoc test for multiple comparisons against the control group. Statistical significance was defined as: ** *p* < 0.01, **** *p* < 0.0001.

## 5. Conclusions

In this study, BA-HP-β-CD and BA-SBE-β-CD were successfully prepared using the solvent stirring method and characterized by solubility and dissolution tests, SEM, XRD, FTIR, and TG-DSC. The results demonstrated that the combination of cyclodextrin inclusion technology and thermosensitive hydrogels provides an efficient and feasible new strategy for the nasal delivery of BA. This dual-carrier system significantly improved the solubility, dissolution rate, cellular permeability, in vitro release behavior, and in vivo pharmacokinetic properties of BA by synergistically leveraging the solubilization and permeation-enhancing effects of CDs, as well as the thermoresponsive and prolonged retention characteristics of the hydrogel matrix. This approach offers a valuable formulation route for the nasal delivery of poorly soluble and poorly permeable drugs and holds promise for the development of new therapeutic formulations.

Future research should focus on validating the therapeutic efficacy and safety of this delivery system in specific disease models. In conclusion, this study not only provides an innovative solution for the nasal delivery of BA but also opens new avenues for the delivery research of poorly soluble drugs, with significant scientific importance and potential application value.

## Figures and Tables

**Table 1:** Orthogonal experimental results for baicalein-hydroxypropyl-β-cyclodextrin inclusion complex (BA-HP-β-CD).

Number	A Molar Ratio of Host to Guest Molecules	B Stirring Time/min	C Amount of Water Added/mL	D Inclusion Temperature/°C	Encapsulation Efficiency, EE/%
1	1:1	60	10	40	15.7
2	1:1	90	20	50	12.5
3	1:1	120	30	60	10.7
4	1:2	60	20	60	32.6
5	1:2	90	30	40	37.6
6	1:2	120	10	50	31.7
7	1:3	60	30	50	64.6
8	1:3	90	10	60	73.2
9	1:3	120	20	40	52.1
K_1_	12.97	37.63	40.2	35.13	
K_2_	33.97	41.10	32.4	36.27	
K_3_	63.30	31.50	37.63	38.83	
R	50.33	9.60	7.80	3.70	

**Table 2:** Orthogonal experimental results for baicalein-sulfobutylether-β-cyclodextrin inclusion complex (BA-SBE-β-CD).

Number	A Molar Ratio of Host to Guest Molecules	B Stirring Time/min	C Amount of Water Added/mL	D Inclusion Temperature/°C	Encapsulation Efficiency, EE/%
1	1:2	60	20	20	15.5
2	1:2	90	30	30	21.2
3	1:2	120	40	40	34.6
4	1:3	60	30	40	16.4
5	1:3	90	40	20	21.1
6	1:3	120	20	30	24.1
7	1:4	60	40	30	89.4
8	1:4	90	20	40	79.4
9	1:4	120	30	20	72.2
K_1_	23.77	40.43	39.67	36.27	
K_2_	20.53	40.57	36.60	44.90	
K_3_	80.33	43.63	48.37	43.47	
R	59.80	3.20	11.77	8.63	

**Table 3:** Solubility results (Mean ± SD, *n* = 3).

Type	Solubility (μg/mL)	RSD (%)
BA	3.08 ± 0.06	2.1
BA-HP-β-CD	1761.36 ± 63.34	3.6
BA-SBE-β-CD	1811.32 ± 56.56	3.1

**Table 4:** Physicochemical properties of baicalein-hydroxypropyl-β-cyclodextrin inclusion complex thermosensitive hydrogel and (BA/HP-Gel) and baicalein-sulfobutylether-β-cyclodextrin inclusion complex thermosensitive hydrogel (BA/SBE-Gel) (Mean ± SD, *n* = 3).

Physicochemical Properties	BA/HP-Gel	BA/SBE-Gel
pH	7.46 ± 0.02	7.38 ± 0.01
Gelation Temperature (°C)	31.1 ± 0.3	31.2 ± 0.3
Gelation Time (s)	45.0 ± 1.7	58.3 ± 1.5
Content (mg/mL)	5.16 ± 0.02	4.67 ± 0.19

**Table 5:** In vitro release pharmacokinetic fitting results of BA, BA/HP-Gel, and BA/SBE-Gel.

Model	BA		BA/HP-Gel		BA/SBE-Gel	
	Fitting Equation	R^2^	Fitting Equation	R^2^	Fitting Equation	R^2^
Zero-order kinetics	Q = 3.4887 + 0.04246 t	0.9458	Q = 10.4948 + 0.12959 t	0.8180	Q = 7.9790 + 0.05954 t	0.8950
First-order kinetics	Q = 16.09985(1 − e^−0.01331t^)	0.7392	Q = 56.1030(1 − e^−0.000943t^)	0.9934	Q = 32.6596(1 − e^−0.01079t^)	0.9759
Higuchi kinetics	Q = 0.9176 t^1/2^ + 0.1680	0.9753	Q = 3.1732 t^1/2^ − 2.786	0.9663	Q = 1.5855 t^1/2^ + 0.9622	0.9776
Ritger-Pappas kinetics	Q = 0.9536 t^0.4976^	0.9741	Q = 2.441 t^0.5336^	0.9576	Q = 2.0316 t^0.4623^	0.9812

**Table 6:** Pharmacokinetic parameters (Mean ± SD, *n* = 4).

Pharmacokinetic Parameters	Units	BA-Gel	BA/HP-Gel	BA/SBE-Gel
T_max_	h	0.12 ± 0.09	0.05 ****	0.05 ****
C_max_	ng/mL	14.09 ± 4.86	750.51 ± 57.56 ****	922.5 ± 52.0 ****
t_1/2_	h	3.46 ± 0.86	13.36 ± 3.84 **	14.56 ± 4.09 **
AUC_last_	h·ng/mL	22.62 ± 12.27	213.26 ± 25.40 ****	226.19 ± 8.18 ****
AUC_0→∞_	h·ng/mL	58.69 ± 3.43	365.92 ± 45.4 **	409.53 ± 38.42 **
MRT_last_	h	1.10 ± 0.31	2.24 ± 0.17	2.13 ± 0.19
F	%	-	623.5	697.8

Note: *p* values from paired-sample *t*-tests comparing the BA/HP-Gel and BA/SBE-Gel groups with the BA-Gel group. ** *p* < 0.01, **** *p* < 0.0001. Parameter definitions: T_max_, time to peak concentration; C_max_, maximum concentration; t_1/2_, elimination half-life; AUC_last_, AUC to last measurable concentration; AUC_0→∞_, total AUC to infinity; MRT_last_, mean residence time to last concentration; F, relative bioavailability compared to a reference formulation.

**Table 7:** Orthogonal design of BA-HP-β-CD.

Level	A (Molar Ratio of Host and Guest Molecules)	B (Stirring Time/min)	C (Water Addition/mL)	D (Inclusion Temperature/°C)
1	1:1	60	10	40
2	1:2	90	20	50
3	1:3	120	30	60

**Table 8:** Orthogonal design of BA-SBE-β-CD.

Level	A (Molar Ratio of Host and Guest Molecules)	B (Stirring Time/min)	C (Water Addition/mL)	D (Inclusion Temperature/°C)
1	1:2	60	20	20
2	1:3	90	30	30
3	1:4	120	40	40

## Data Availability

The original contributions presented in this study are included in the article. Further inquiries can be directed to the corresponding author.

## Supplementary Materials

The following supporting information can be downloaded at: https://www.mdpi.com/article/10.3390/ph19050781/s1, Table S1: Precision test results for baicalein plasma samples (Mean ± SD, *n* = 5); Table S2: Results of matrix effect and extraction recovery tests for baicalein rat plasma samples (Mean ± SD, *n* = 5); Table S3: Results of dilution effect tests for baicalein plasma samples (Mean ± SD, *n* = 5); Table S4: Results of stability tests for baicalein plasma samples (Mean ± SD, *n* = 5); Table S5: Factors and levels of the Box–Behnken response surface design; Table S6: Response surface experimental design and results; Table S7: Analysis of the regression model equation.

## Abbreviations

The following abbreviations are used in this manuscript:

ATCC	American type culture collection
BA	Baicalein
BA-HP-β-CD	Baicalein-hydroxypropyl-β-cyclodextrin inclusion complex
BA-SBE-β-CD	Baicalein-sulfobutylether-β-cyclodextrin inclusion complex
BA/HP-Gel	Baicalein-hydroxypropyl-β-cyclodextrin inclusion complex thermosensitive hydrogel
BA/SBE-Gel	Baicalein-sulfobutylether-β-cyclodextrin inclusion complex thermosensitive hydrogel
BCS	Biopharmaceutics classification system
CCK-8	Cell counting kit-8
CD	Cyclodextrin
CS	Chitosan
DSC	Differential scanning calorimetry
EE	Encapsulation efficiency
FTIR	Fourier transform infrared spectroscopy
HP-β-CD	Hydroxypropyl-β-cyclodextrin
HPLC	High-performance liquid chromatography
MDCK	Madin-Darby canine kidney
PM	Physical mixtures
SBE-β-CD	Sulfobutyl-β-cyclodextrin
SD	Standard deviation
SEM	Scanning electron microscope
TEER	Trans-epithelial electrical resistance
TG-DSC	Thermogravimetric analysis-differential scanning calorimetry
UHPLC-MS/MS	Ultra-high performance liquid chromatography-tandem mass spectrometry
XRD	X-ray diffraction
β-GP	pentahydrate-β-glycerophosphate sodium
AUC	Area under the plasma concentration-time curve
C_max_	Maximum concentration
F	Relative bioavailability
MRT	Mean residence time
t_1/2_	Elimination half-life
T_max_	Time to peak concentration

## References

[1] Tuli H.S., Aggarwal V., Kaur J., Aggarwal D., Parashar G., Parashar N.C., Tuorkey M., Kaur G., Savla R., Sak K. (2020). Baicalein: A metabolite with promising antineoplastic activity. Life Sci..

[2] Oo A., Teoh B.T., Sam S.S., Bakar S.A., Zandi K. (2019). Baicalein and baicalin as Zika virus inhibitors. Arch. Virol..

[3] Yang S., Wang H., Yang Y., Wang R., Wang Y., Wu C., Du G. (2019). Baicalein administered in the subacute phase ameliorates ischemia-reperfusion-induced brain injury by reducing neuroinflammation and neuronal damage. Biomed. Pharmacother..

[4] Li W., Pi J., Zhang Y., Ma X., Zhang B., Wang S., Qi D., Li N., Guo P., Liu Z. (2018). A strategy to improve the oral availability of baicalein: The baicalein-theophylline cocrystal. Fitoterapia.

[5] Davis M.E., Brewster M.E. (2004). Cyclodextrin-based pharmaceutics: Past, present and future. Nat. Rev. Drug Discov..

[6] da Silva Júnior W.F., de Oliveira Pinheiro J.G., Moreira C.D., de Souza F.J., de Lima Á.A. (2017). Alternative technologies to improve solubility and stability of poorly water-soluble drugs. Multifunctional Systems for Combined Delivery, Biosensing and Diagnostics.

[7] Liu Z., Ye L., Xi J., Wang J., Feng Z.-g. (2021). Cyclodextrin polymers: Structure, synthesis, and use as drug carriers. Prog. Polym. Sci..

[8] Malanga M., Szemán J., Fenyvesi É., Puskás I., Csabai K., Gyémánt G., Fenyvesi F., Szente L. (2016). “Back to the future”: A new look at hydroxypropyl beta-cyclodextrins. J. Pharm. Sci..

[9] Stolzke T., Krieg F., Peng T., Zhang H., Häusler O., Brandenbusch C. (2022). Hydroxylpropyl-β-cyclodextrin as potential excipient to prevent stress-induced aggregation in liquid protein formulations. Molecules.

[10] Dai K., Wu J., Liu X., Wang S., Liu Y., Li H., Wang H. (2024). Inclusion complex of quercetin with sulfobutylether β-cyclodextrin: Preparation, characterization, antioxidant and antibacterial activities and the inclusion mechanism. RSC Adv..

[11] Wang Z., Zhang Q., Luo L., Sun T., Guo X. (2017). Comparison of three S-β-CDs with different degrees of substitution for the chiral separation of 12 drugs in capillary electrophoresis. Chirality.

[12] Cid-Samamed A., Rakmai J., Mejuto J.C., Simal-Gandara J., Astray G. (2022). Cyclodextrins inclusion complex: Preparation methods, analytical techniques and food industry applications. Food Chem..

[13] Qiu N., Zhao X., Liu Q., Shen B., Liu J., Li X., An L. (2019). Inclusion complex of emodin with hydroxypropyl-β-cyclodextrin: Preparation, physicochemical and biological properties. J. Mol. Liq..

[14] Rosaini H., Sandra N., Halim A. (2021). Characterization of celecoxib β-cyclodextrin inclusion complexes using solvent evaporation method. Asian J. Pharm. Res. Dev..

[15] Duchêne D., Bochot A. (2016). Thirty years with cyclodextrins. Int. J. Pharm..

[16] Jansook P., Ogawa N., Loftsson T. (2018). Cyclodextrins: Structure, physicochemical properties and pharmaceutical applications. Int. J. Pharm..

[17] Varan G., Varan C., Erdoğar N., Hıncal A.A., Bilensoy E. (2017). Amphiphilic cyclodextrin nanoparticles. Int. J. Pharm..

[18] Arya P., Raghav N. (2021). In-vitro studies of Curcumin-β-cyclodextrin inclusion complex as sustained release system. J. Mol. Struct..

[19] Savjani K.T., Gajjar A.K., Savjani J.K. (2012). Drug solubility: Importance and enhancement techniques. Int. Sch. Res. Not..

[20] Grassin-Delyle S., Buenestado A., Naline E., Faisy C., Blouquit-Laye S., Couderc L.-J., Le Guen M., Fischler M., Devillier P. (2012). Intranasal drug delivery: An efficient and non-invasive route for systemic administration: Focus on opioids. Pharmacol. Ther..

[21] Patel M.R., Patel R.B., Bhatt K.K., Patel B.G., Gaikwad R.V. (2016). Paliperidone microemulsion for nose-to-brain targeted drug delivery system: Pharmacodynamic and pharmacokinetic evaluation. Drug Deliv..

[22] Dreiss C.A. (2020). Hydrogel design strategies for drug delivery. Curr. Opin. Colloid Interface Sci..

[23] Ho T.-C., Chang C.-C., Chan H.-P., Chung T.-W., Shu C.-W., Chuang K.-P., Duh T.-H., Yang M.-H., Tyan Y.-C. (2022). Hydrogels: Properties and applications in biomedicine. Molecules.

[24] Thang N.H., Chien T.B., Cuong D.X. (2023). Polymer-based hydrogels applied in drug delivery: An overview. Gels.

[25] Mikhail A.S., Morhard R., Mauda-Havakuk M., Kassin M., Arrichiello A., Wood B.J. (2023). Hydrogel drug delivery systems for minimally invasive local immunotherapy of cancer. Adv. Drug Deliv. Rev..

[26] Liu Y., Zhu M., Meng M., Wang Q., Wang Y., Lei Y., Zhang Y., Weng L., Chen X. (2023). A dual-responsive hyaluronic acid nanocomposite hydrogel drug delivery system for overcoming multiple drug resistance. Chin. Chem. Lett..

[27] Yang X., Zhang C., Deng D., Gu Y., Wang H., Zhong Q. (2022). Multiple stimuli-responsive MXene-based hydrogel as intelligent drug delivery carriers for deep chronic wound healing. Small.

[28] Wang L., Luo Y., Song Y., He X., Xu T., Zhang X. (2024). Hydrogel-functionalized bandages with Janus wettability for efficient unidirectional drug delivery and wound care. ACS Nano.

[29] Xiang C., Zhang X., Zhang J., Chen W., Li X., Wei X., Li P. (2022). A porous hydrogel with high mechanical strength and biocompatibility for bone tissue engineering. J. Funct. Biomater..

[30] Sánchez-Machado D.I., López-Cervantes J., Correa-Murrieta M.A., Sánchez-Duarte R.G., Cruz-Flores P., de la Mora-López G.S., Nabavi S.M. (2019). Chitosan. Nonvitamin and Nonmineral Nutritional Supplements.

[31] Xu J., Chang L., Xiong Y., Peng Q. (2024). Chitosan-based hydrogels as antibacterial/antioxidant/anti-inflammation multifunctional dressings for chronic wound healing. Adv. Healthc. Mater..

[32] Peers S., Montembault A., Ladaviere C. (2022). Chitosan hydrogels incorporating colloids for sustained drug delivery. Carbohydr. Polym..

[33] Deng A., Kang X., Zhang J., Yang Y., Yang S. (2017). Enhanced gelation of chitosan/β-sodium glycerophosphate thermosensitive hydrogel with sodium bicarbonate and biocompatibility evaluated. Mater. Sci. Eng. C.

[34] Ammar H., Salama H., Ghorab M., Mahmoud A. (2006). Implication of inclusion complexation of glimepiride in cyclodextrin–polymer systems on its dissolution, stability and therapeutic efficacy. Int. J. Pharm..

[35] Zhou Q., Wei X., Dou W., Chou G., Wang Z. (2013). Preparation and characterization of inclusion complexes formed between baicalein and cyclodextrins. Carbohydr. Polym..

[36] Qiang Y., Wei H., Huang B., Chi H., Fu J. (2024). Inclusion complex of turmeric essential oil with hydroxypropyl-β-cyclodextrin: Preparation, characterization and release kinetics. Curr. Res. Food Sci..

[37] Li Q., Pu H., Tang P., Tang B., Sun Q., Li H. (2018). Propyl gallate/cyclodextrin supramolecular complexes with enhanced solubility and radical scavenging capacity. Food Chem..

[38] Hou W., Guo W., Dai Z., Ren H., Luo X., Fu J. (2025). Baicalin nanofiber membranes: A comprehensive study on preparation, characterization, and antimicrobial pharmacological activities. J. Mol. Struct..

[39] Chao J., Wang X., Xu M., Zuo Y. (2019). Characterization and enhanced antioxidant activity of the inclusion complexes of baicalin with p-sulfonatocalix [n] arenes. J. Incl. Phenom. Macrocycl. Chem..

[40] Xu Y., Wang X., Fu Z., Huang J., Lai X., Tai Z., Wu X. (2025). Enhanced Solubility, Stability, and Safety through Busulfan/Sulfobutyl Ether β-Cyclodextrin Inclusion Complexes. ACS Appl. Polym. Mater..

[41] Tian L., Wu Y., Hou Y., Dong Y., Ni K., Guo M. (2024). Environmentally friendly UV absorbers: Synthetic characterization and biosecurity studies of the host–guest supramolecular complex. Int. J. Mol. Sci..

[42] Tang P., Li S., Wang L., Yang H., Yan J., Li H. (2015). Inclusion complexes of chlorzoxazone with β-and hydroxypropyl-β-cyclodextrin: Characterization, dissolution, and cytotoxicity. Carbohydr. Polym..

[43] Liang B., Hao J., Zhu N., Han L., Song L., Hong H. (2023). Formulation of nitrendipine/hydroxypropyl-β-cyclodextrin inclusion complex as a drug delivery system to enhance the solubility and bioavailability by supercritical fluid technology. Eur. Polym. J..

[44] Hu X., Wang X., Han L., Li S., Zhou W. (2021). Antioxidant and antimicrobial polyvinyl alcohol electrospun nanofibers containing baicalein-hydroxypropyl-β-cyclodextrin inclusion complex. Colloids Surf. A Physicochem. Eng. Asp..

[45] Ren L., Wang J., Chen G. (2019). Preparation, optimization of the inclusion complex of glaucocalyxin A with sulfobutylether-β-cyclodextrin and antitumor study. Drug Deliv..

[46] Du J., Zhao L., Yang S., Huang Y., Chi S., Ruan Q., Zheng P., Hu R., Zhao Y. (2019). Preparation, characterization, solubilization and antioxidant activity of polyamine modified β-cyclodextrins with baicalein inclusion complexes. J. Incl. Phenom. Macrocycl. Chem..

[47] Yang J., Chen A., He X., Lu S. (2023). Fabrication of baicalein-encapsulated zeolitic imidazole framework as a novel nanocomposited wound closure material to persuade pH-responsive healing efficacy in post-caesarean section wound care. Int. Wound J..

[48] Xu L., Jiang W., Ni H., Xu W., Li A., Wang J., Zhu J., Chen S., Tong S. (2025). An efficient strategy for preparation of sulfobutylether-β-cyclodextrin chiral stationary phases for liquid chromatography. J. Chromatogr. A.

[49] Zhu W., Wu J., Guo X., Sun X., Li Q., Wang J., Chen L. (2020). Development and physicochemical characterization of chitosan hydrochloride/sulfobutyl ether-β-cyclodextrin nanoparticles for cinnamaldehyde entrapment. J. Food Biochem..

[50] Fang J., He Y., Cao Y., Shi Y., Wang H., Hong Z., Chai Y. (2023). Effect of P-Glycoprotein on the Blood–Brain Barrier Transport of the Major Active Constituents of Salvia miltiorrhiza Based on the MDCK-MDR1 Cell Model. ACS Chem. Neurosci..

[51] Li L., Tan L., Zhang Q., Cheng Y., Liu Y., Li R., Hou S. (2023). Nose-to-brain delivery of self-assembled curcumin-lactoferrin nanoparticles: Characterization, neuroprotective effect and in vivo pharmacokinetic study. Front. Bioeng. Biotechnol..

[52] Furubayashi T., Inoue D., Nishiyama N., Tanaka A., Yutani R., Kimura S., Katsumi H., Yamamoto A., Sakane T. (2020). Comparison of various cell lines and three-dimensional mucociliary tissue model systems to estimate drug permeability using an in vitro transport study to predict nasal drug absorption in rats. Pharmaceutics.

[53] Gholizadeh H., Cheng S., Pozzoli M., Messerotti E., Traini D., Young P., Kourmatzis A., Ong H.X. (2019). Smart thermosensitive chitosan hydrogel for nasal delivery of ibuprofen to treat neurological disorders. Expert Opin. Drug Deliv..

[54] Yu L., Tian Y., Ding Y., Chi Z., Liu C. (2024). Chitosan/β-glycerophosphate porous microsphere prepared by facile water-in-water emulsion as a topical hemostatic material. Int. J. Biol. Macromol..

[55] Huang C.-L., Chen Y.-B., Lo Y.-L., Lin Y.-H. (2016). Development of chitosan/β-glycerophosphate/glycerol hydrogel as a thermosensitive coupling agent. Carbohydr. Polym..

[56] Dang Q., Liu K., Zhang Z., Liu C., Liu X., Xin Y., Cheng X., Xu T., Cha D., Fan B. (2017). Fabrication and evaluation of thermosensitive chitosan/collagen/α, β-glycerophosphate hydrogels for tissue regeneration. Carbohydr. Polym..

[57] Bai L., Xu X.M., He J., Pan S.Z. (2009). Inclusion complexation, encapsulation interaction and inclusion number in cyclodextrin chemistry. Coord. Chem. Rev..

[58] Samineni R., Chimakurthy J., Konidala S. (2022). Emerging role of biopharmaceutical classification and biopharmaceutical drug disposition system in dosage form development: A systematic review. Turk. J. Pharm. Sci..

[59] Khadka P., Ro J., Kim H., Kim I., Kim J.T., Kim H., Cho J.M., Yun G., Lee J. (2014). Pharmaceutical particle technologies: An approach to improve drug solubility, dissolution and bioavailability. Asian J. Pharm. Sci..

[60] Savolainen M., Kogermann K., Heinz A., Aaltonen J., Peltonen L., Strachan C., Yliruusi J. (2009). Better understanding of dissolution behaviour of amorphous drugs by in situ solid-state analysis using Raman spectroscopy. Eur. J. Pharm. Biopharm..

[61] Aiassa V., Garnero C., Zoppi A., Longhi M.R. (2023). Cyclodextrins and their derivatives as drug stability modifiers. Pharmaceuticals.

[62] Davis S.S., Illum L. (2003). Absorption enhancers for nasal drug delivery. Clin. Pharmacokinet..

[63] Merkus F., Verhoef J., Marttin E., Romeijn S., Van der Kuy P., Hermens W., Schipper N. (1999). Cyclodextrins in nasal drug delivery. Adv. Drug Deliv. Rev..

[64] Rassu G., Sorrenti M., Catenacci L., Pavan B., Ferraro L., Gavini E., Bonferoni M.C., Giunchedi P., Dalpiaz A. (2021). Versatile nasal application of cyclodextrins: Excipients and/or actives?. Pharmaceutics.

[65] D’Souza S. (2014). A review of in vitro drug release test methods for nano-sized dosage forms. Adv. Pharm..

[66] Modi S., Anderson B.D. (2013). Determination of drug release kinetics from nanoparticles: Overcoming pitfalls of the dynamic dialysis method. Mol. Pharm..

[67] Jing Q., Jiang X.-G. (2021). Preparation and evaluation of intranasal in situ gel of Bupleuri Radix volatile oil and baicalin. J. Sichuan Univ. (Med. Sci.).

[68] Zhou J.-F., Duan L., Wang Y.-X., Wang C.-L., Tian M.-L., Qi X.-J., Qiu F. (2022). Design, characterization of Resveratrol-Thermosensitive Hydrogel System (Res-THS) and evaluation of its anti-depressant effect via intranasal administration. Mater. Des..

[69] González N.N., Rassu G., Cossu M., Catenacci L., Sorrenti M.L., Cama E.S., Serri C., Giunchedi P., Gavini E. (2024). A thermosensitive chitosan hydrogel: An attempt for the nasal delivery of dimethyl fumarate. Int. J. Biol. Macromol..

[70] Yu Y., Cheng Y., Tong J., Zhang L., Wei Y., Tian M. (2021). Recent advances in thermo-sensitive hydrogels for drug delivery. J. Mater. Chem. B.

[71] Mura P., Maestrelli F., Cirri M., Mennini N. (2022). Multiple roles of chitosan in mucosal drug delivery: An updated review. Mar. Drugs.

[72] Rahmanian-Devin P., Baradaran Rahimi V., Askari V.R. (2021). Thermosensitive chitosan-β-glycerophosphate hydrogels as targeted drug delivery systems: An overview on preparation and their applications. Adv. Pharmacol. Pharm. Sci..

[73] Ojeda-Hernández D.D., Velasco-Lozano S., Fraile J.M., Mateos-Díaz J.C., Rojo F.J., Benito-Martín M.S., Selma-Calvo B., de la Fuente-Martín S., García-Martín M., Larriba-González M.T. (2024). Thermosensitive chitosan-based hydrogel: A vehicle for overcoming the limitations of nose-to-brain cell therapy. Acta Biomater..

[74] Gizurarson S. (2015). The effect of cilia and the mucociliary clearance on successful drug delivery. Biol. Pharm. Bull..

[75] Varga P., Németh A., Zeiringer S., Roblegg E., Budai-Szűcs M., Balla-Bartos C., Ambrus R. (2024). Formulation and investigation of differently charged β-cyclodextrin-based meloxicam potassium containing nasal powders. Eur. J. Pharm. Sci..

[76] Ahad A., Bin Jardan Y.A., Raish M., Al-Mohizea A.M., Al-Jenoobi F.I. (2022). Hydroxypropyl-β-cyclodextrin for delivery of sinapic acid via inclusion complex prepared by solvent evaporation method. Processes.

[77] Chang C., Song M., Ma M., Song J., Cao F., Qin Q. (2023). Preparation, characterization and molecular dynamics simulation of rutin–cyclodextrin inclusion complexes. Molecules.

[78] dos Santos Lima B., de Alcântara Campos C., da Silva Santos A.C.R., Santos V.C.N., Trindade G.d.G.G., Shanmugam S., Pereira E.W.M., Marreto R.N., Duarte M.C., da Silva Almeida J.R.G. (2019). Development of morin/hydroxypropyl-β-cyclodextrin inclusion complex: Enhancement of bioavailability, antihyperalgesic and anti-inflammatory effects. Food Chem. Toxicol..

[79] Wang Y., Li X., Yan C., Xie L., Yang Y. (2023). Baicalin exhibits a protective effect against cisplatin-induced cytotoxic damage in canine renal tubular epithelial cells. Metabolites.

[80] Fedi A., Vitale C., Ponschin G., Ayehunie S., Fato M., Scaglione S. (2021). In vitro models replicating the human intestinal epithelium for absorption and metabolism studies: A systematic review. J. Control. Release.

[81] Bhuiyan M.H., Clarkson A.N., Ali M.A. (2023). Optimization of thermoresponsive chitosan/β-glycerophosphate hydrogels for injectable neural tissue engineering application. Colloids Surf. B Biointerfaces.

[82] Do N.H., Pham T.H., Le P.K., Ha A.C. (2023). Thermo-responsive Chitosan/β-glycerophosphate hydrogels directly post-loading anti-inflammatory diclofenac sodium. J. Sol-Gel Sci. Technol..

[83] Inoue D., Yamashita A., To H. (2022). Development of in vitro evaluation system for assessing drug dissolution considering physiological environment in nasal cavity. Pharmaceutics.

