# Supplementary material for: Baicalein-Cyclodextrin Inclusion Complexes Nasal Thermosensitive Hydrogel: Bioavailability Improvement and Pharmacokinetic Evaluation in Rats
Source: Pharmaceuticals (Basel). 2026 May 16;19(5):781. doi: 10.3390/ph19050781 (PMC13210136; doi:10.3390/ph19050781)
Supplement: Supplementary file 1 [file pharmaceuticals-19-00781-s001.zip › pharmaceuticals-4275293-supplementary.pdf]

Validation of UHPLC–MS/MS Method

Precision and Accuracy

According to the linear range of the standard curve, baicalein plasma samples at low, medium, and high concentrations (30, 150, and 375 ng/mL) were prepared. These samples were processed following the same procedure and analyzed five times within a single day to assess intra-day precision, and once daily for five consecutive days to assess inter-day precision.

Matrix Effect and Extraction Recovery

Three concentrations of baicalein plasma samples at low, medium, and high levels (30, 150, and 375 ng/mL) were prepared using rat blank plasma. After pretreatment according to the plasma sample protocol, the samples were injected, and the peak area was recorded as A_1. Acetonitrile was used instead of blank plasma, while the remaining operations were the same as described above, and the peak area was recorded as A_2. Subsequently, 50 μL of blank plasma was taken, protein was precipitated with acetonitrile, and the supernatant was collected by centrifugation. Then, baicalein reference solution and internal standard solution were added to prepare three concentrations of baicalein plasma samples at low, medium, and high levels (30, 150, and 375 ng/mL), and the peak area was recorded as A_3.

Matrix Effect (%) = A_1 / A_2 × 100%

Extraction Recovery (%) = A_1 / A_3 × 100%

Each concentration was determined in quintuplicate.

Dilution Effect

Five groups of blank plasma were taken and prepared into plasma samples with a concentration of 10 μg/mL in parallel. 5 μL of this plasma sample was mixed with 45 μL of blank plasma and vortexed for 5 minutes to obtain a sample with a concentration of 1 μg/mL. Then 25 μL of the 1 μg/mL plasma sample was mixed with 25 μL of blank plasma and vortexed for 5 minutes to obtain a sample with a concentration of 0.5 μg/mL. The plasma samples were pretreated and then determined for precision and accuracy.

Stability

Rat blank plasma was used to prepare three concentrations of baicalein plasma samples at low, medium and high levels (30, 150, 375 ng/mL). The samples were stored at room temperature for 4 h, at 4 °C for 12 h, subjected to 3 freeze-thaw cycles, and stored at −80°C for 7, 14 and 30 d respectively, then determined and the stability of the plasma samples was calculated. Each group of tests was performed in quintuplicate.

Table S1. Precision test results for baicalein plasma samples (Mean ± SD, n = 5)

Quality Control Concentration (ng/mL)	intra-day precision		Inter-day precision	
	Measured concentration (ng/mL)	RSD (%)	Measured concentration (ng/mL)	RSD (%)
30	32.66 ± 0.30	0.91	32.42 ± 0.84	2.59
150	152.43 ± 1.10	0.72	153.17 ± 3.12	2.04
375	380.58 ± 1.68	0.44	385.11 ± 7.12	1.85

Table S2. Results of matrix effect and extraction recovery tests for baicalein rat plasma samples (Mean ± SD, n = 5)

Quality Control Concentration (ng/mL)	Matrix Effect (%)		Extraction Recovery (%)	
	Mean ± SD	RSD	Mean ± SD	RSD
30	104.71 ± 7.31	6.98	104.23 ± 0.91	0.87
150	107.38 ± 1.82	1.69	102.95 ± 3.58	3.47
375	107.55 ± 0.65	0.60	101.63 ± 0.71	0.70

Table S3. Results of dilution effect tests for baicalein plasma samples (Mean ± SD, n = 5)

Quality Control Concentration (ng/mL)	Measured concentration (ng/mL)	RSD (%)
500	499.41 ± 3.54	0.71

Table S4. Results of stability tests for baicalein plasma samples (Mean ± SD, n = 5)

Quality Control Concentration (ng/mL)	Room temperature for 4 h	At 4°C for 12 h	Three freeze-thaw cycles	Stored at −80°C for 7 d	Stored at −80°C for 14 d	Stored at −80°C for 30 d
30	33.88 ± 0.76	32.09 ± 1.11	32.68 ± 1.47	33.43 ± 1.13	30.42 ± 1.19	33.54 ± 1.02
150	157.59 ± 2.86	152.78 ± 1.59	162.06 ± 4.42	150.73 ± 5.44	150.72 ± 6.23	158.42 ± 5.41
375	387.65 ± 4.81	379.48 ± 3.30	386.06 ± 5.50	380.44 ± 2.95	365.27 ± 14.71	389.88 ± 7.58

Table S5. Factors and levels of the Box-Behnken response surface design

Factor	Level		
	-1	0	1
A.CS% (w/v)	1.6	1.8	2.0
B.β-GP% (w/v)	52	56	60
C.β-GP:CS	3:7	2:3	1:1

Table S6. Response surface experimental design and results

std	Factor 1<br>A.CS (%)	Factor 2<br>B.β-GP (%)	Factor 3<br>C.β-GP:CS	Response<br>Temperature (°C)
1	1.8	56	4:6	29.7
2	2.0	56	3:7	30.9
3	1.8	56	4:6	29.2
4	2.0	56	5:5	24.2
5	1.8	56	4:6	28.7
6	1.8	52	5:5	27.9
7	1.8	56	4:6	29.9
8	1.8	52	3:7	36.8
9	1.6	60	4:6	32.9
10	1.6	52	4:6	33.3
11	1.8	60	5:5	25.6
12	2.0	60	4:6	26.5
13	1.6	56	3:7	39.1
14	2.0	52	4:6	28.1
15	1.6	56	5:5	28.6
16	1.8	60	3:7	34.6
17	1.8	56	4:6	29.5

The Design Expert software was used to perform multivariate regression fitting on the response surface test model. The multiple regression equation for the comprehensive score (Y) with respect to the concentration of CS (A), β-GP (B), and the volume ratio of β-GP to CS (C) is as follows: $Y = +29.4 - 3.02A - 0.8125B - 4.39C - 0.30AB + 0.95AC - 0.025BC + 0.1375A^2 + 0.6625B^2 + 1.16C^2$.

Table S7. Analysis of the regression model equation

Source	Sum of Squares	df	Mean Square	F-value	*P*-value
Model	244.60	9	27.18	102.98	<0.0001
A-CS	73.20	1	73.20	277.37	<0.0001
B-β-GP	5.28	1	5.28	20.01	0.0029
C-β-GP:CS	154.00	1	154.00	583.50	<0.0001
AB	0.3600	1	0.3600	1.36	0.2811
AC	3.61	1	3.61	13.68	0.0077
BC	0.0025	1	0.0025	0.0095	0.9252
A^2	0.0796	1	0.0796	0.3016	0.5999
B^2	1.85	1	1.85	7.00	0.0331
C^2	5.69	1	5.69	21.56	0.0024
Residual	1.85	7	0.2639		
Lack of Fit	0.9675	3	0.3225	1.47	0.3503
Pure Error	0.8800	4	0.2200		
Cor Total	246.45	16			

$R^2 = 0.9925$, Adjusted $R^2 = 0.9829$, Predicted $R^2 = 0.9316$
